# Expression of Calcification and Extracellular Matrix Genes in the Cardiovascular System of the Healthy Domestic Sheep (*Ovis aries*)

**DOI:** 10.3389/fgene.2020.00919

**Published:** 2020-09-08

**Authors:** Hiu-Gwen Tsang, Emily L. Clark, Greg R. Markby, Stephen J. Bush, David A. Hume, Brendan M. Corcoran, Vicky E. MacRae, Kim M. Summers

**Affiliations:** ^1^The Roslin Institute and R(D)SVS, The University of Edinburgh, Edinburgh, United Kingdom; ^2^Nuffield Department of Clinical Medicine, John Radcliffe Hospital, University of Oxford, Oxford, United Kingdom; ^3^Mater Research Institute-University of Queensland, Translational Research Institute, Woolloongabba, QLD, Australia; ^4^The Royal (Dick) School of Veterinary Studies, The University of Edinburgh, Edinburgh, United Kingdom

**Keywords:** sheep, cardiovascular system, extra cellular matrix, gene expression, RNA-seq, network analysis, ectopic calcification

## Abstract

The maintenance of a healthy cardiovascular system requires expression of genes that contribute to essential biological activities and repression of those that are associated with functions likely to be detrimental to cardiovascular homeostasis. Vascular calcification is a major disruption to cardiovascular homeostasis, where tissues of the cardiovascular system undergo ectopic calcification and consequent dysfunction, but little is known about the expression of calcification genes in the healthy cardiovascular system. Large animal models are of increasing importance in cardiovascular disease research as they demonstrate more similar cardiovascular features (in terms of anatomy, physiology and size) to humans than do rodent species. We used RNA sequencing results from the sheep, which has been utilized extensively to examine calcification of prosthetic cardiac valves, to explore the transcriptome of the heart and cardiac valves in this large animal, in particular looking at expression of calcification and extracellular matrix genes. We then examined genes implicated in the process of vascular calcification in a wide array of cardiovascular tissues and across multiple developmental stages, using RT-qPCR. Our results demonstrate that there is a balance between genes that promote and those that suppress mineralization during development and across cardiovascular tissues. We show extensive expression of genes encoding proteins involved in formation and maintenance of the extracellular matrix in cardiovascular tissues, and high expression of hematopoietic genes in the cardiac valves. Our analysis will support future research into the functions of implicated genes in the development of valve calcification, and increase the utility of the sheep as a large animal model for understanding ectopic calcification in cardiovascular disease. This study provides a foundation to explore the transcriptome of the developing cardiovascular system and is a valuable resource for the fields of mammalian genomics and cardiovascular research.

## Introduction

The cardiovascular system plays a crucial role not only in the distribution of nutrients to the various cells, tissues and organs within the mammalian body, but also in the removal of waste products. Extensive regulatory mechanisms are required to support this functional system, with perturbations likely to lead to abnormalities, and thus give rise to cardiovascular-related disease, a major cause of morbidity and mortality worldwide, with an estimated 17.3 to 17.5 million deaths per year (Townsend et al., 2016; World Health Organisation, 2017). Cardiac valvulopathies are becoming increasingly prevalent in the aging population (Nkomo et al., 2006). A recent United Kingdom study of nearly 80,000 adult patients referred for echocardiography found that 50% had some degree of cardiac valve dysfunction (Marciniak et al., 2017). In contrast, only 12% of the same patient group had left ventricular systolic dysfunction.

The maintenance of a healthy cardiovascular system requires expression of genes that contribute to essential biological activities and repression of those that are associated with functions likely to be detrimental to cardiovascular homeostasis. A major pathological process that disrupts cardiovascular homeostasis is ectopic calcification, which is associated with aging, hypertension and atherosclerosis (Abedin et al., 2004; Towler, 2008; Zhu et al., 2012; Tsang et al., 2016). Ectopic calcification results from abnormal mineral metabolism, involving the deposition of calcium phosphate, in the form of hydroxyapatite (HA). In cardiovascular tissues, ectopic calcification most critically affects the arteries and cardiac valves, and is a significant, independent risk factor of cardiovascular mortality (Giachelli, 2004; Li et al., 2006; Zhu et al., 2012). Most individuals above 60 years of age have gradually enlarging calcium deposits in their major arteries (Allison et al., 2004; Demer and Tintut, 2008). Ectopic calcification is a highly regulated, active process involving a variety of signaling pathways, with evidence suggesting the involvement of mechanisms similarly observed in bone formation (Boström et al., 1993; Lanzer et al., 2014). However, the exact molecular basis underpinning the complex process of ectopic calcification, particularly the dysregulated expression of genes involved in cardiovascular function, has yet to be fully defined.

A number of factors have been implicated in the phenotypic transition of vascular smooth muscle cells (VSMCs) into osteocytic-, osteoblastic-, and chondrocytic-like cells (Yang et al., 2004; Li et al., 2006; Giachelli, 2009; Zhu et al., 2011,2012). These include increase in osteochondrogenic markers, including TNAP (*ALPL* gene), osteopontin (also known as secreted phosphoprotein 1; *SPP1* gene), and the transcription factor RUNX2 (Steitz et al., 2001; Rajamannan et al., 2003; Lomashvili et al., 2004; Speer et al., 2009; Yang et al., 2009; Zhu et al., 2012), as well as decrease in mineralization inhibitors, including ENPP1, MGP, ecto-5′-nucleotidase NT5E (also known as cluster of differentiation 73, CD73), and FBN1 (Luo et al., 1997; Schurgers et al., 2008; Rutsch et al., 2011; St. Hilaire et al., 2011; Albright et al., 2015). During the calcification process VSMCs enter a synthetic state with abundant production of extracellular matrix (ECM) proteins (Hruska et al., 2005) followed by matrix vesicle-mediated calcification (Giachelli, 2009; Leopold, 2015). Indeed recent comparative transcription profiling has identified over 50 ECM genes identically regulated by calcifying VSMCs and bone-forming osteoblasts (Alves et al., 2014), with ECM proteins likely acting in concert with each other to determine the extent of calcification. A similar process is likely followed by cardiac valve cells undergoing calcification. Nevertheless, although the sequence of events leading to normal bone mineralization is better understood, the specific mechanisms by which ectopic calcification occurs remain ambiguous, as affected cells may still retain their overall identity, despite acquiring osteoblastic properties (Frink, 2002; Zhu et al., 2012; Alves et al., 2014).

In recent decades, both non-invasive and invasive therapies for cardiovascular disease have advanced considerably. This advancement has been underpinned by basic research, with animal models being of key importance. Of growing value is the use of large animal models of cardiovascular disease (reviewed in Tsang et al., 2016). Sheep and pigs, for example, are more similar in their cardiovascular features (in terms of anatomy, genetics, physiology, and size) to humans than are rodent species. Evidence suggests there are significant phenotypic differences between mouse and human stem cells (Ginis et al., 2004; Gabdoulline et al., 2015) and large animals might therefore provide greater similarity to humans at the cellular and molecular level. Early developmental stages can be studied in detail in large animal models, which is limited in scope in both human and mouse (Emmert et al., 2013). Finer resolution of regions of the cardiovascular system is also possible with the increased size of the heart and vessels of the large animal models. Sufficient RNA for transcriptomic studies can be obtained from a single animal, so that inter-animal variability can be assessed. The major benefit of large animals in cardiovascular disease (CVD) clinical research remains, however, their application in the development of interventional technologies and implantable devices (reviewed in Tsang et al., 2016). In particular, sheep have been used extensively to model the outcomes of prosthetic cardiac valve implantation (Barnhart et al., 1982; Schoen et al., 1994; Flameng et al., 2006; Baraki et al., 2009; Fukunishi et al., 2016; Bonetti et al., 2019). These studies revealed extensive calcification of the prosthetic valves, which was ameliorated in decellularized prostheses. Cell culture models using sheep aortic valve interstitial cells and vascular smooth muscle cells have shown that the cultures mineralize under calcifying conditions (Nigam and Srivastava, 2009; Shimizu et al., 2011; Tsang et al., 2018). Characterizing the normal transcriptome of the healthy mammalian cardiovascular system will allow better understanding of the cellular changes induced by these treatments.

The increasing use of high-throughput technologies such as RNA sequencing (RNA-seq), has been continually expanding the number of gene expression datasets available for specific tissues and cells. Although there are various public resources, the transcriptomic data available for the mammalian cardiovascular system are generally limited to the “heart” or ventricular tissue, such as in BioGPS^1^ and the Expression Atlas online database (EMBL-EBI)^2^. In the human GTex Project (Mele et al., 2015), for example, two cardiovascular tissues are included (Heart – Atrial Appendage and Heart – Left Ventricle) from a large number of individuals (*n* = 372 and *n* = 386 respectively). RNA-seq has been used to greatly enhance resolution of cardiovascular disease in humans (reviewed in Wirka et al., 2018) and generate baseline estimates of gene expression in developing cardiovascular tissues (Pervolaraki et al., 2018). However, comparable resources were not available for large animal models, to aid in the development of interventions and other treatments for cardiovascular disease.

This study describes gene expression in the mammalian cardiovascular system, supporting and extending the high resolution gene expression atlas for sheep (Clark et al., 2017). Initially we present tissue-specific gene expression profiles in the heart muscle and cardiac valves using RNA-seq. We then use reverse transcriptase quantitative PCR (RT-qPCR) to measure myocardial and arterial tissue gene expression during development in the sheep focusing on inhibitors of calcification in the healthy cardiovascular system.

## Materials and Methods

### Sample Collection

Five developmental stages from Texel x Scottish Blackface sheep were analyzed: 100-day gestation (fetal), newborn, 1 week, 8 weeks and 2 years (*n* = 3–6 per group). The adult samples were from three male and three female adult Texel × Scottish Blackface sheep at 2 years of age (*n* = 6 in total) described previously (Clark et al., 2017). Samples were collected within an hour and 30 min post euthanasia. 17 different tissues were collected from adults; the equivalent tissues were collected from fetuses at day 100 of gestation and young lambs where possible. Detailed dissection of tissues was performed by the same two researchers, for all sheep, in order to standardize tissue sampling. Details of the samples included in each of the three sets of analyses described herein (RNA-seq of eight tissues, developmental stage expression profiles and calcification gene expression profiles) are included in Table 1. After dissection, tissues of interest were placed into RNAlater (Thermo Fisher Scientific) and stored according to the manufacturer’s instructions. RNA was extracted from tissues using TRIzol (Thermo Fisher Scientific) as described in Clark et al. (2017). RNA integrity (RIN^e^) was estimated on an Agilent 2200 Tapestation System (Agilent Genomics).

**TABLE 1:** Details of the samples, number of biological replicates, and developmental stage of all samples included in the analyses.

Tissue	No. of replicates	Developmental stage
**RNA-seq**		
Aortic Valve	4	Adult (2 years)
Left AV Valve	4	Adult (2 years)
Right AV Valve	4	Adult (2 years)
Left Auricle	4	Adult (2 years)
Right Auricle	5	Adult (2 years)
Left Ventricle	6	Adult (2 years)
Right Ventricle	6	Adult (2 years)
Skeletal Muscle (Bicep)	6	Adult (2 years)
**RT-qPCR (developmental stages)**		
Left Ventricle	3–5	Fetus d100, newborn, 8 weeks, 2 years
Interventricular Septum	3–5	Fetus d100, newborn, 1 week, 8 weeks, 2 years
Aortic Root	3–5	Newborn, 1 week, 8 weeks, 2 years
Aortic Arch	3–5	Newborn, one week, 2 years
Abdominal Aorta	3–5	Newborn, 8 weeks, 2 years
Pulmonary Artery	3–5	Newborn, 8 weeks, 2 years
**RT-qPCR (calcification genes)**		
Left Auricle	6	Adult (2 years)
Left Atrium	6	Adult (2 years)
Left Ventricle	6	Adult (2 years)
Right Auricle	6	Adult (2 years)
Right Atrium	6	Adult (2 years)
Right Ventricle	6	Adult (2 years)
Interventricular Septum	6	Adult (2 years)
Cranial Vena Cava	6	Adult (2 years)
Aortic Valve	6	Adult (2 years)
Left AV Valve	6	Adult (2 years)
Right AV Valve	6	Adult (2 years)
Pulmonary Valve	6	Adult (2 years)
Aortic Base	6	Adult (2 years)
Aortic Arch	6	Adult (2 years)
Descending Thoracic Aorta	6	Adult (2 years)
Abdominal Aorta	6	Adult (2 years)
Pulmonary Artery	6	Adult (2 years)

*AV, atrioventricular.*

### RNA-seq

The RNA-seq analysis we present in this manuscript is based on a subset of data, from seven cardiovascular tissues and one skeletal muscle (Table 1), from our high resolution atlas of gene expression for domestic sheep (Clark et al., 2017). All procedures were described in Clark et al. (2017). After quality control a small number of samples, despite multiple extraction attempts, were of insufficient quality for RNA-seq, and as such some of the tissues in the present analysis have less than six biological replicates (Supplementary Table S1). RNA-seq libraries were generated and sequenced by Edinburgh Genomics (Edinburgh, United Kingdom). Expression was quantified using the alignment-free transcript quantification tool Kallisto v0.43.0 (Bray et al., 2016). Kallisto generates expression level estimates orders of magnitude faster than previous approaches and so was ideally suited for processing the large volumes of data constituting the expression atlas described previously (Clark et al., 2017). However, it was contingent on the user providing a robust set of reference transcripts as input. To obtain transcript models that were absent from the original sheep annotation (Oar v3.1) we performed genome-guided *de novo* assembly using the HISAT/StringTie ‘new Tuxedo’ protocol (Pertea et al., 2016), as previously described (Clark et al., 2017). The raw RNA-seq data are deposited in the European Nucleotide Archive (ENA) under study accession number PRJEB19199^3^. Supplementary Table S1 provides the details of the cardiovascular tissues included in the sheep atlas dataset and analyzed further here. It was necessary to normalize these estimates according to the methods described in Bush et al. (2017) to account for the two different library types (ribo-depleted total RNA for left ventricle and poly-A selected mRNA for all other tissues). The gene expression estimates for the sheep gene expression atlas dataset are publicly available on BioGPS^4^, and we have included the gene expression estimates for the subset of tissues re-analyzed here as Supplementary Dataset S1.

### Network Analysis of the Gene Expression Estimates From RNA-seq

Expression estimates for each gene were analyzed using the network visualization tool, BioLayout^5^ (Theocharidis et al., 2009; Livigni et al., 2018). Expression values were averaged across biological replicates for each tissue. To minimize spurious correlations due to low expression noise, only genes with expression > 1 TPM in at least one averaged sample were included in the analysis. Similarities between individual gene expression profiles were determined by the calculation of a Pearson pairwise correlation matrix for both sample-to-sample and gene-to-gene comparisons. The co-expression network was laid out using the Fruchterman–Rheingold algorithm (Fruchterman and Reingold, 1991). The dataset was filtered to remove relationships where the Pearson correlation coefficient (which is the statistical measure of the strength of a linear relationship between paired data) was below a threshold of *r* ≥ 0.91 (sample-to-sample) and *r* ≥ 0.99 (gene-to-gene). The Markov clustering algorithm (MCL) was applied at an inflation value (which determines cluster granularity) of 2.2 (van Dongen and Abreu-Goodger, 2012) to identify groups of transcripts with closely related expression patterns. Clusters were numbered in order of decreasing cluster size. The online Database for Annotation, Visualization and Integrated Discovery (DAVID) Functional Annotation tool^6^ was used for Gene Ontology (GO) analysis.

### Functional Annotation of Unannotated Genes

In spite of the extensive annotation process undertaken for the original sheep atlas [summarized above and described in detail in Clark et al. (2017)], we identified a number of unannotated or poorly annotated genes with no or minimal associated GO terms and previously no known function. A summary of the numbers of minimally annotated genes in the clusters discussed in this paper is presented in Supplementary Table S2. Protein-coding genes that contribute to common generic and cell-specific cellular processes or pathways generally form co-expression clusters, allowing the inference of the function of a gene of previously unknown function (Oliver, 2000; Freeman et al., 2012; Klomp and Furge, 2012; Carpanini et al., 2017). Using this ‘guilt-by-association’ principle we were able to provide functional annotation information for genes that were expressed in cardiovascular tissues and previously unannotated in Oar v3.1, based on the average expression pattern of the cluster in which they were found.

### Reverse Transcriptase Quantitative PCR (RT-qPCR)

RNA samples (collected and purified as described above) with RIN^e^ > 7 were used for RT-qPCR. RT-qPCR reactions were performed using PrecisionPLUS-MX-SY Mastermix (containing SYBR Green; Primerdesign Ltd) following the manufacturer’s protocol. Details of the twenty-four sheep-specific primers used are listed in Table 2. Because of the limitations of the sheep genome sequence it was not possible to design primers for all genes of interest. Primers were designed using the current version of the sheep genome Oar v3.1^7^ with Primer3 software^8^ to span exon–exon junctions, and obtained from Invitrogen (Paisley, United Kingdom) or Primerdesign Ltd (Eastleigh, United Kingdom). In addition to those in Table 2, primers were designed for *FBN3, ABCC6, SOST, ALPL* (2 sets of primers), *AHSG, CA2, TRIM24, ADIPOQ, SMAD6, MCP1/CCL2, TNF, IL1R1, FN1, MMP9, TIMP2* but were not used for further analysis, based on cost, time constraints, expression level during preliminary testing and optimization of the RT-qPCR. In addition, we were unable to analyze a number of established calcification genes, as certain genes, including *ALPL*, are only up-regulated in tissues undergoing pathological calcification and not detectable in healthy tissue. RT-qPCR was carried out on three technical replicates for each sample. Gene expression was normalized to the geomean of *GAPDH* and *YWHAZ*. Normalized data were expressed as 2-ΔCt values from the 2-ΔΔCt method (Livak and Schmittgen, 2001), and transformed using the natural log (Log_e_).

**TABLE 2:** Sheep primers for RT-qPCR.

Gene	Category	Forward primer (5′-3′)	Reverse primer (5′-3′)
*ADAMTS6*; ADAM metallopeptidase with thrombospondin type 1 motif 6	ECM	AGGTGTATGATGCCGATGAACA	CTGCGGGAATACTGTTGGTGA
*ANKH*; Progressive ankylosis protein	Calcification inhibitor	AGTTCACGTTCGTCTGCATG	TGGAACCGGGAAGAAGGAAA
*BGLAP*; bone gamma-carboxyglutamate protein	Calcification	GCCTGGTGATGCAGAGTCG	GCTCCAGCGGATCTGGGTA
*BGN*; Biglycan	ECM	GGAGAACAGCGGCTTTTGAAC	GAGGGTCTCAGGGAGGTCTT
*COL1A1*; Collagen type I alpha 1	ECM	AAGGAGACACTGGTGCCAAG	GCCAGCAGGTCCAGGTTC
*COL1A2*; Collagen type I alpha 2	ECM	TGGTCAGACTGGTCCTGCT	CTGTGGTCCAACAACTCCTCT
*COL3A1*; Collagen type III alpha 1	ECM	GCTGTTGACGGAGGATGCT	ATTATGTCATCACAGAGAACGGATC
*DKK3*; Dickkopf WNT signaling pathway inhibitor 3	Calcification inhibitor	Not available (Primerdesign Ltd)	Not available (Primerdesign Ltd)
*ENPP1*; Ectonucleotide pyrophosphatase/phosphodiesterase 1	Calcification inhibitor	CCCAGACTCCCTTACAGTGT	GATCCGAGCTCTGTGTAGCT
*FBN1*; Fibrillin 1	ECM, calcification inhibitor	GCTGCCAGAACATCATCGG	CTGTTCGTATTGGAAGCCGG
*FBN2*; Fibrillin 2	ECM	CTGGGAGGCTACAGGTGTG	GACGAGCACTCATTCACGTC
*FMOD*; fibromodullin	ECM	GAGGAAGACTCTCACTGGTGG	TGGAGAGCCGTAGGCGTAA
*GAPDH*; glyceraldehyde 3-phosphate dehydrogenase	Reference gene	Not available (Primerdesign Ltd)	Not available (Primerdesign Ltd)
*MGP*; matrix Gla protein	Calcification inhibitor	ACAACAGAGATGGAGAGCGA	CGGAAATAACGGTCGTAGGC
*MMP2*; matrix metalloproteinase 2	ECM	ACAAATTCTGGAGATACAATGAGGT	CAGGTCCACCACAGCATCC
*NPPA*; natriuretic peptide A	Vascular remodeling	Not available (Primerdesign Ltd)	Not available (Primerdesign Ltd)
*NT5E*; ecto-5′-nucleotidase (also known as CD73)	Calcification inhibitor	TCCTTGTCAGTGGTGGAGAC	GCAGAAAACTGGATCCGACC
*RUNX2*; Runt-related transcription factor 2	Calcification	CTCCTCCATCCATCCACTCC	CAGAGGCAGAAGTCAGAGGT
*SLC20A1* (*PiT1*); sodium-dependent phosphate transporter 1	Calcification	ACATCTTGAACGCCGCTA	AGTAGCAGCAATAGCAGTGGTA
*SMAD2*; SMAD family member 2	Calcification inhibitor	GGGAGGAGTGAGGAGTGCTC	GGTTTCCTGGTTTAGCTCTCA
*SPP1*; osteopontin, also known as secreted phosphoprotein 1)	Calcification	TGACCCATCTCAGAAGCAGA	CTCGGCCATCATTTGTGCTT
*TIMP1*; TIMP metallopeptidase inhibitor 1	ECM	GCCTTATACCAGCGTTATGAGAT	GCAGGGGTGTAGATGAATCG
*TNFRSF11B*; osteoprotegerin	Calcification inhibitor	GGAGGCGTTCTTCAGGTTTG	CGGCAAGCTTTCCATCAACT
*YWHAZ*; tyrosine 3-mono-oxygenase	Reference gene	Not available (Primerdesign Ltd)	Not available (Primerdesign Ltd)

*Primers in black were designed using Primer3 (http://primer3.ut.ee/) to span exon–exon junctions, and obtained from Invitrogen (Paisley, United Kingdom). Primers in blue were obtained from Primerdesign Ltd. (Eastleigh, United Kingdom).*

### Statistical Analyses

Statistical analyses of RT-qPCR results were performed using Minitab 17 (Coventry, United Kingdom). The Kolmogorov–Smirnov normality test was performed to check whether experimental data were normally distributed. In this study, one-way analysis of variance (ANOVA) using a general linear model incorporating Fisher’s least significant difference (LSD) method was used for pairwise comparisons. RT-qPCR gene expression data in this study are expressed as mean ± standard deviation (SD), and *p*-value < 0.05 from the ANOVA (which allows for multiple comparisons) was considered significant. Dotplots were made in R v3.2.2^9^, using the R package ‘ggplot2’ with error bars showing mean ± SD. Individual data points are also included in the dotplots.

## Results

### Gene Expression Profiles Reflect Anatomical Structure

**FIGURE 1:**
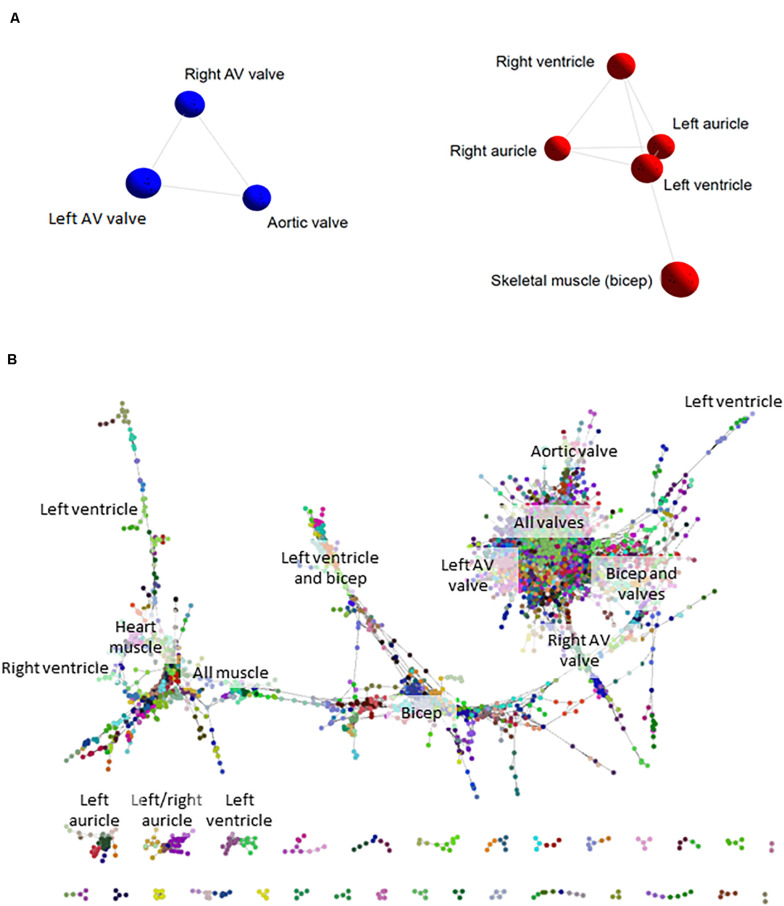
Cardiovascular gene co-expression networks. **(A)** Sample-to-sample analysis. Network layout of 8 tissue types revealed two distinct components: the first containing cardiac valve samples (blue), and the second with myocardium/skeletal muscle tissues (red). Pearson correlation co-efficient *r* ≥ 0.91. **(B)** Gene-to-gene analysis. Circles represent genes and lines correlation between them of *r* ≥ 0.99. Circles of the same color were allocated to the same expression cluster by Markov clustering algorithm (MCL) clustering of the graph (inflation value 2.2). The labels indicate the tissues where clusters of genes were most highly expressed. The gene-to-gene network was comprised of 11,341 nodes (genes) with 938,652 connections and resulted in 555 clusters containing > 3 genes (the figure shows only the larger elements).

We initially took advantage of the RNA-seq data from the sheep atlas project (Clark et al., 2017) to compare the transcriptomes of skeletal muscle, heart and cardiac valves. The network visualization generated by BioLayout for sample-to-sample analysis is similar to a principal components analysis and creates a network graph where the tissue samples (averaged across biological replicates from up to 6 adult sheep; Supplementary Dataset S1) with highly similar expression profiles are located close together. The resultant graph contained all 8 nodes (tissue samples) that were connected by 10 edges (connections between nodes at a correlation coefficient of ≥ 0.91; Figure 1A). Two distinct elements were identified: an element containing the five myocardium/skeletal muscle samples and a cardiac valve element (red and blue respectively in Figure 1A). The network indicated that there were close similarities in the overall expression profiles of genes in skeletal muscle and heart muscle, which were distinct from the cardiac valve tissues. Similar grouping of skeletal muscle and heart muscle tissues was previously observed by Lukk et al. (2010).

### Tissue-Specific Expression Clusters in the Cardiovascular System

Network-based gene-to-gene analysis of Supplementary Dataset S1 grouped genes according to their expression pattern across the muscle and valve samples, producing a gene co-expression network (GCN) (Gaiteri et al., 2014). A high correlation coefficient of *r* ≥ 0.99 was necessary to discriminate expression patterns due to the similarity of expression in the relatively small set of samples being analyzed. The resultant graph (Figure 1B) included 11,341 nodes (genes) with 938,652 edges (correlations at *r* ≥ 0.99 between them). There was one main extended element containing the majority of the genes and 331 smaller elements of less than 30 genes. MCL clustering of the graph (inflation = 2.2) resulted in 555 clusters containing > 3 genes. We focused on 143 clusters with ≥10 genes. All genes with high expression in one or more of the cardiac valves were close together in the network (Figure 1B), and there were also regions containing genes with high expression in all muscle, bicep muscle, heart muscle and the different heart chambers, as indicated in Figure 1B. Details of the genes in the clusters and average expression profiles are included in Supplementary Dataset S2. Some clusters (where the difference in average expression was less than 3-fold; 88 clusters) were considered ubiquitous and generally contained housekeeping genes. For 36 of the 143 clusters, expression was at least 3-fold higher in the three valve samples than in the heart and skeletal muscle. Seven of these showed high expression only in aortic valve while four were highest in the right atrioventricular (tricuspid) valve and 3 were higher in the left atrioventricular (mitral) valve. Several clusters contained genes that were higher in skeletal muscle than cardiac muscle or valves, and some were high in the myocardium samples only. Two clusters contained genes that were high only in the skeletal (bicuspid) muscle and left ventricle. There was no cluster of genes that were high in the ventricles alone, or in a single myocardium sample, but two clusters contained genes that were higher in the auricles than in the other tissues sampled. Interestingly there were some clusters of genes with higher expression in the skeletal muscle and heart valves than the myocardium or with higher expression in the skeletal muscle and left ventricle (Supplementary Dataset S2). Expression profiles of a subset of ECM, VC and heart function genes are shown in Supplementary Figure S1. Table 3 summarizes the expression patterns of four clusters with higher expression in the cardiac valves and one with highest expression in the heart auricles. These clusters were chosen because they showed at least 3-fold difference in average expression among samples and contained genes relating to the ECM and calcification. They are discussed below along with representative clusters showing higher expression in the heart or biceps muscle samples.

**TABLE 3:** Summary of five clusters from the sheep cardiovascular transcriptome dataset.

Cluster	Number of genes	Number of un-annotated genes	Expression profile description	Functional class	GO term	EASE score (*p*-value)	EASE score (*p*-value; Benjamini corrected)	Genes included (Gene symbols)
1	3543	529	Cardiac valves	Various, Housekeeping	* (BP) mRNA 3′-end processing; RNA export from nucleus	^a,b^8.1E-6; ^a,b^1.5E-4	^a,b^0.0064; ^a^0.063, ^b^0.055	COL1A1, COL3A1, MMP2, TIMP1
3	192	42	Cardiac valves (highest in aortic valve)	ECM organization, bone development	(BP) extracellular matrix organization; skeletal system development; osteoblast differentiation	6.02E-4; 0.00336; 1.12E-5	0.124; 0.31; 0.0099	BGN, COL1A2, SPARC; BGLAP, COL1A2, GDF10; BMP4, BGLAP, SNAI1-2, SOX8
22	27	5	Cardiac valves	Housekeeping, Immune	(BP) immune response (MF) integral component of membrane	0.067; 0.061	0.999; 0.928	CCR6, ENPP1, NFIL3; CD47, ENPP1, IL6ST, SMAD2
24	25	3	Cardiac valves (Aortic valve > Left AV valve > Right AV valve)	ECM	(BP) positive regulation of transcription from RNA polymerase II promoter; transcription from RNA polymerase II promoter (CC) extracellular matrix	0.108; 0.123; 0.045	0.999; 0.999; 0.943	MEOX1, TRPS1, RBPJ, NLRP3; FMOD, FBN1, COL6A1
36	20	5	Auricles (Left > Right)	Muscle contraction	(BP) potassium ion transport; (MF) calcium ion binding; (CC) extracellular space	0.002; 0.06; 0.221	0.16; 0.921; 0.999	KCNQ3, KCNJ3, KCNK3; MYL7, PAM, MYL4; DKK3, NPPA, PAM

Gene Ontology (GO) term analysis was performed using DAVID Functional Annotation tool (https://david.ncifcrf.gov/). AV, atrioventricular; BP, biological processes; CC, cellular component; MF: molecular function. Full gene lists and cardiovascular expression data are presented in Supplementary Dataset S1. * Up to 3000 genes maximum can be input into DAVID, thus two runs were performed: ^*a*^GO term significant in top 3000 genes; ^*b*^GO term significant in bottom 3000 genes. EASE score = modified Fisher Exact. > indicates decreasing expression.

**FIGURE 2:**
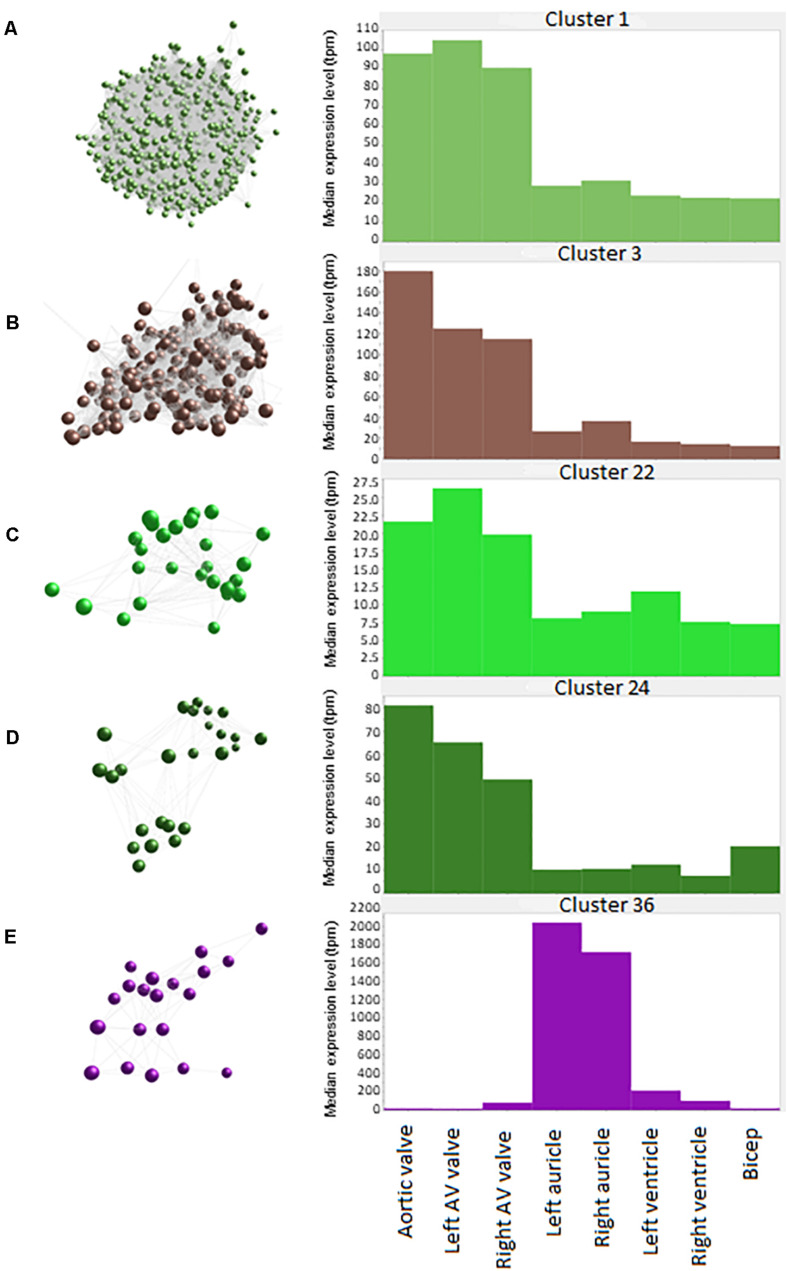
Average expression of genes within clusters. Spheres (nodes) in co-expression clusters (left) denote individual genes; lines represent connections between genes. The histograms (right) show median expression levels in transcripts per million (TPM) in the different tissues. *X* axis shows the samples; *Y* axis shows the average expression for the cluster in TPM. AV, atrioventricular. **(A)** Cluster 1, the largest cluster, contained 3543 genes, with 529 unannotated genes. A co-expression cluster highly expressed in the sheep cardiac valves compared to the myocardium and bicep. **(B)** Cluster 3 contained 192 genes, with 40 unannotated genes. A co-expression cluster highly expressed in the sheep cardiac valves, particularly in the aortic valves, compared to the myocardium and bicep. **(C)** Cluster 22 contained 27 genes, with 5 unannotated genes. A co-expression cluster highly expressed in the sheep cardiac valves compared to the myocardium and bicep. **(D)** Cluster 24 contained 25 genes, with 3 unannotated genes. A co-expression cluster highly expressed in the sheep cardiac valves compared to the myocardium and bicep. **(E)** Cluster 36 contained 20 genes, with 5 unannotated genes. A co-expression cluster highly expressed in the sheep auricles compared to the cardiac valves, the ventricles and the bicep.

Cluster 1 contained 3543 genes (Figure 2A). Genes in this cluster showed approximately a 3-fold greater expression in the cardiac valves than in the other tissues. A wide variety of genes was included in this cluster. The cluster contained genes enriched for GO terms associated with cell structure, such as *COL1A1* (encoding collagen type I alpha 1) and *COL3A1* (collagen type III alpha 1), *MMP*2, -*9*, -*19*, -*20*, and -*28* (matrix metalloproteinases), *FBN2* (fibrillin-2) and *TIMP1* (tissue inhibitor of matrix metalloproteinases 1) (Table 3). There were also multiple genes expressed specifically by macrophages in sheep (Clark et al., 2017) and other species (Fantom Consortium and the Riken PMI and CLST (DGT), 2014; Lizio et al., 2015; Summers et al., 2020), including *CSF1R, AIF1*, *SPI1* and *CSF2RA/B* and a number of interleukin and interferon responsive genes. In addition, members of the smoothened signaling pathway (*SMO, GLI1-3*) involved in cilium formation and function, genes associated with RNA transcription and processing (for example POLR genes) and some genes associated with cell proliferation (for example centromere protein genes) were in this cluster. Cluster 1 also contained genes associated with TGF beta signaling, including *TGFB3, TGFBI, TGFBR2*, and *TGFBR3.*

Cluster 3 (Figure 2B) contained 192 genes with highest expression in the cardiac valves, particularly the aortic valve, at approximately 4.5 to 18-fold higher than the myocardial and skeletal muscle tissues. This cluster was enriched for GO terms associated with extracellular matrix organization, skeletal system development, osteoblast differentiation and cartilage morphogenesis. Genes in this cluster included those encoding bone morphogenetic protein 4 (*BMP4*), collagen type I alpha 2 (*COL1A2*) and bone gamma-carboxyglutamate protein (*BGLAP*, also known as osteocalcin) (Table 3). Other valve specific genes in this cluster included *MYH10* (myosin heavy chain 10), Potassium channel gene *KCNU1* and *BGN* (ECM protein biglycan) which was 15- to 20-fold higher in the valves. Genes encoding various transcription factors were also contained within this cluster, including FOS like 2, AP-1 transcription factor subunit (*FOSL2*), Twist basic helix-loop-helix transcription factor 2 (*TWIST2*), Snail family transcriptional repressor 1 and 2 (*SNAI1* and *SNAI2*) and Sry homeobox 8 (*SOX8*). In cluster 3, 42 genes were unannotated and not included in the input into DAVID.

Like cluster 1, the 27 genes in cluster 22 (Figure 2C) also showed relatively ubiquitous expression, with smaller differential between the cardiac valves and the myocardium and skeletal muscle than those in cluster 1 (approximately 3-fold difference) (Figure 2C). Genes in cluster 22 included *ENPP1* (ectonucleotide pyrophosphate/phosphodiesterase 1), *ADAMTS6* (ADAM metallopeptidase with thrombospondin type 1 motif 6) and *SMAD2* (SMAD family member 2). Some of the genes in this cluster are annotated as immune-related, including C-C motif chemokine receptor 6 (*CCR6*), interleukin6 signal transducer (*IL6ST*) and nuclear factor, interleukin 3 (*NFIL3*) (Table 3) but these genes are less specific to hematopoietic cells than those in Cluster 1 (Clark et al., 2017; BioGPS, 2020).

The 25 genes in cluster 24 (Figure 2D) were also more highly expressed in the cardiac valves, at up to 16-fold higher than the myocardium, and approximately 4 to 5-fold higher than the bicep (representing skeletal muscle). Genes encoding proteins involved in transcriptional regulation were included in this cluster, including mesenchyme homeobox 1 (*MEOX1*), the GATA-regulated gene repressor transcriptional repressor GATA binding 1 (*TRPS1*) and recombination signal binding protein for immunoglobulin kappa J region (*RBPJ*) (Table 3). ECM protein encoding genes were also included, such as *FBN1* (fibrillin-1), *FMOD* (fibromodulin), and *COL6A1* (collagen type VI alpha 1) as well as the macrophage specific gene NLR family pyrin domain containing 3 (*NLRP3*) (Table 3).

The 20 genes in cluster 36 (Figure 2E) showed high expression in the auricles, at up to 2,000-fold higher than the other tissues. Genes included in this cluster were involved in muscle contraction through potassium ion transport (Table 3), e.g., potassium voltage-gated channel subfamily Q member 3 (*KCNQ3*), subfamily J member 3 (*KCNJ3*) and subfamily K member 3 (*KCNK3*), as well as peptidylglycine alpha-amidating monooxygenase (*PAM*) and myosin light chains 4 and 7 (*MYL4 and -7*) (Table 3). Other genes to note include natriuretic peptide A (*NPPA*) and Dickkopf WNT signaling pathway inhibitor 3 (*DKK3*) (Table 3). Examination of the wider sheep gene expression atlas^10^ showed that many of the genes in this cluster were also highly expressed in brain regions, consistent with the function of many as ion transport channels.

A number of clusters contained genes that were high in both bicep (representing skeletal muscle) and the heart regions and 2- to 3-fold lower in the valves. As might be expected, these clusters were enriched for genes involved in mitochondrial function, reflecting the energy requirements of muscle tissue. In contrast, Cluster 4 (164 nodes) expression was high only in bicep and contained genes specific to skeletal muscle such as a range of troponin and myosin genes, the ryanodine receptor 1 gene (*RYR1*) and sodium, potassium and calcium ion channel genes. Other bicep-specific clusters included Clusters 23 (25 genes) and 28 (24 genes). These three clusters were comprised of genes encoding proteins for zinc-dependent proteases, e.g., *ADAMTS20* (ADAM metallopeptidase with thrombospondin type 1 motif 20) and genes involved in actin binding and motor activity, e.g., *MYH15* (myosin heavy chain 15). A small proportion of clusters exhibited expression patterns that were specific to skeletal muscle (bicep) and left ventricle. The largest of these clusters was cluster 13 (48 genes), which included genes encoding proteins involved in hydrolysis of extracellular nucleotides, e.g., ectonucleotide pyrophosphatase/phosphodiesterase 3 (*ENPP3*) and transcription factors, e.g., caudal type homeobox 1 (*CDX1*) and solute carriers, e.g., *SLC16A4* and *SLC26A3*. Several smaller clusters, 66 (15 genes), 83 (13 genes) and 90 (13 genes), exhibited left ventricle specific expression profiles and were comprised of genes with a similar function to those within cluster 13.

In summary, the cardiac valves showed more consistent expression of ECM genes, while both valves and the muscle samples had detectable levels of many transcripts associated with resident macrophage populations. The transcriptome of cardiac muscle was similar to skeletal muscle in this analysis, although some tissue specific gene expression could be seen. For example, the potassium channel genes *KCNJ3, KCNK3*, and *KCNQ3*, the myosin light chain genes *MYL4* and *MYL7* and a natriuretic peptide gene, *NPPA* were auricle-specific (Cluster 36).

### Functional Annotation of Unannotated Genes

Each cluster contained a number of genes which had no informative gene name. These included genes with no GO terms, those described as pseudogenes and those encoding uncharacterized proteins. There were also a number of genes with homology to human open reading frames of unknown function or with homology to uncharacterized transmembrane protein genes and other poorly annotated gene families. A summary of the minimally annotated genes in the clusters discussed above is provided in Supplementary Table S2. While many of these genes had low expression, some were highly expressed. For example, *ENSOARG00000020353* had a maximum of nearly 6,000 TPM in aortic valve. It is described as a novel gene with a 24 amino acid match to parathymosin (*PTMS*) in the bovine. According to the ‘guilt by association’ principle (Oliver, 2000) since this gene was found within a cluster of genes with high expression in the cardiac valve samples it may well have a similar function to other annotated genes that are co-expressed in Cluster 1. Other examples include *ENSOARG00000005484* in Cluster 36, which was expressed at around 18 TPM in left and right auricle. This gene has some homology to *FAM155A* and *FAM155B* (*Tmem28* in mouse), probably a transmembrane calcium ion transporter (UniProtKB B1AL88 and O75949 respectively). Further exploration of the 923 ENSOARG genes that were included in the cluster analysis should allow attribution of putative functions based on their presence in a cluster of genes of known function.

### Myocardial Tissue Gene Expression During Development

**FIGURE 3:**
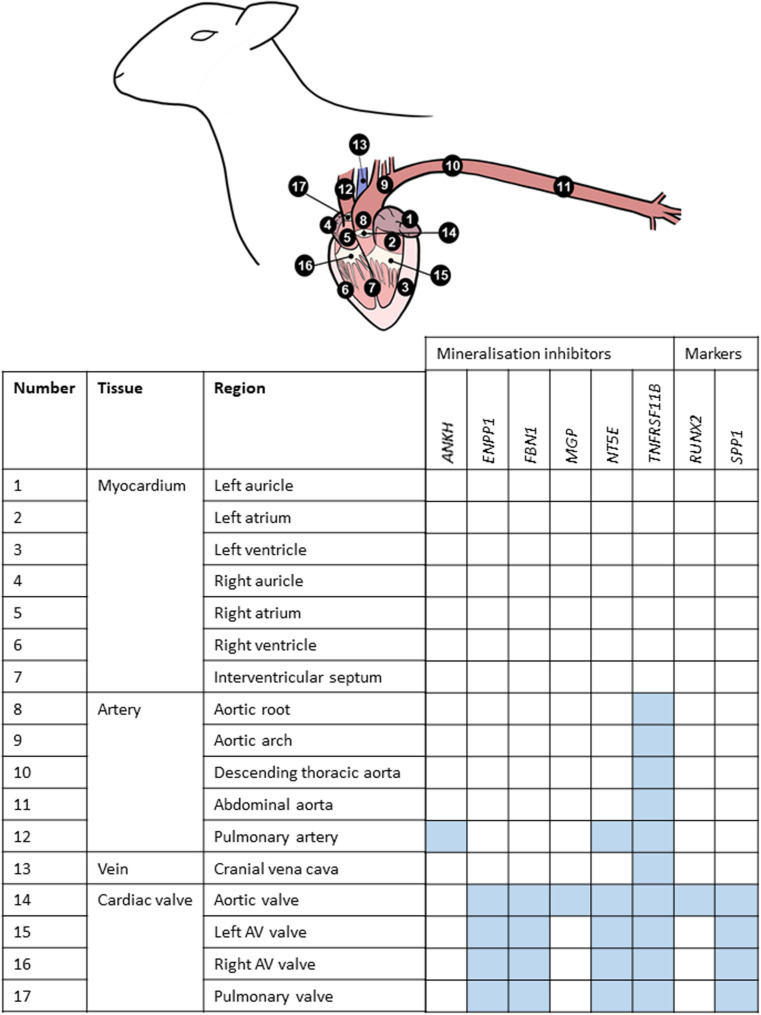
Summary of mRNA expression profiles of key vascular calcification genes in the sheep cardiovascular system. Blue blocks indicate where genes were found to be most highly expressed in this study. AV, atrioventricular.

To explore the gene expression differences in the whole cardiovascular system, we used RT-qPCR to analyze selected genes involved in extracellular matrix (ECM) composition and in maintenance of calcium homeostasis, in a range of samples from pre- and post-natal developmental stages of the sheep. A number of genes which are the focus of studies in our group because they are associated with cardiovascular pathology and/or ectopic calcification were examined. The genes and the diseases associated with them are listed in Supplementary Table S3. The RT-qPCR results for all genes analyzed including standard deviations are included in Supplementary Dataset S3. A summary of the results for the developmental stages is presented in Table 4 and the full results with significance levels can be seen in Supplementary Figures S2–S7. Results for different tissues in the adult sheep are summarized in Figure 3 and full results with significance levels can be found in Supplementary Figures S8–S10. Below we highlight the differences in expression for selected genes. All differences reported below were significant at *p* < 0.05 by ANOVA analysis.

**TABLE 4:** Summary of expression profiles of ECM and calcification genes during pre- to post- natal development in the sheep cardiovascular system.

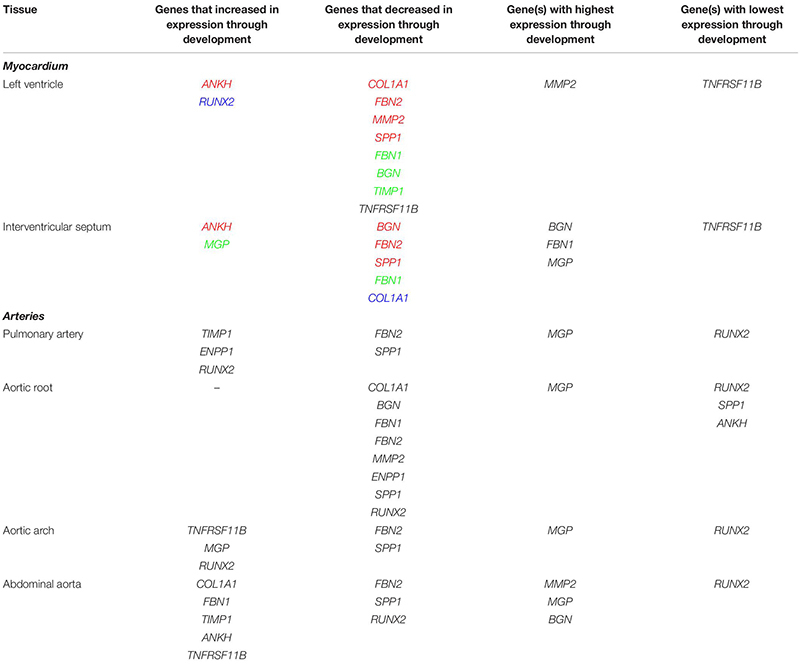

*Color key for myocardial tissue only (no fetal samples were available for arterial tissues): Red – Overall changed expression from fetal to adult; Green – Changed expression from pre- to post- natal stages; Blue – Changed expression during post-natal stages.*

In the left ventricle free wall, the ECM protein-encoding genes showed significant decreases in relative expression during development from 100 days gestation to 2 years of age, although the timing varied, with *BGN* and *TIMP1* declining before birth while *COL1A1, MMP2* and *FBN2* decreased after birth. *FBN1* dropped rapidly before birth but then increased at 8 weeks (Table 4 and Supplementary Figure S2). The expression of *SPP1* (also known as *OPN*), which promotes calcification, decreased overall with age while *RUNX2*, which is also involved in mineralization increased during development. The mineralization inhibitor *ANKH* showed increases in relative mRNA expression, while *TNFRSF11B* (also known as *OPG* and thought to restrict calcification) showed a significant decrease in its expression levels after birth. Overall, the expression levels of *RUNX2* and *TNFRSF11B* were low compared to the other tested genes and *MMP2, BGN* and *MGP* were the highest compared to the other genes in the left ventricle (Table 4 and Supplementary Figure S2).

In contrast, in the interventricular septum, the ECM genes *COL1A1, BGN* and *MMP2* were largely unchanged during development while *FBN1* and *FBN2* exhibited decreases in mRNA expression as development progressed (Table 4 and Supplementary Figure S3). In general, *SPP1* expression decreased with age in the interventricular septum although higher expression was observed in the 1 week old lambs compared to newborn lambs (*p* < 0.05). As in the left ventricle, the expression of *ANKH* showed an increasing trend with age, but with a significant increase between the fetal and newborn lamb samples (*p* < 0.05). Overall, the expression of *MGP* (mineralization inhibitor) did not change, although the 1 week old lambs showed statistically significant higher expression compared to the fetal lambs (*p* < 0.05). Genes that were most highly expressed in the interventricular septum were *BGN, MGP* and *FBN1*, whereas *TNFRSF11B* showed the lowest levels of expression within this tissue (Table 4).

### Arterial Tissue Expression During Development

It was not possible to obtain samples from the fetal animals for the arteries, but we examined gene expression changes from newborn to adult. In the pulmonary artery, *FBN2* expression decreased and *TIMP1* expression increased between birth and 2 years of age (Table 4 and Supplementary Figure S4). Expression of both *RUNX2* and *ENPP1* (likely to have opposing effects on mineralization) was significantly higher in the 2-year old sheep (*p* < 0.01; Table 4 and Supplementary Figure S4). *SPP1* was significantly lower in the 2-year old sheep compared to both newborn and 8-week old lambs (*p* < 0.01). In the pulmonary artery, *MGP* was the most highly expressed of the genes tested, followed by *BGN, MMP2, ENPP1* and *COL1A1*, with *RUNX2* as the gene with the lowest expression levels (Table 4).

In the aortic root, all the significant changes involved a decrease from young lambs to 2-year-old adults. Both the ECM protein genes (*COL1A1, BGN, MMP2, FBN1*, and *FBN2*) and the genes with opposing effects on mineralization (*ENPP1* and *SPP1)* showed decreases in their expression (Table 4), and were significantly lower at 2 years of age compared to newborn and 1 week old lambs (Supplementary Figure S5). Overall, in the aortic root, *MGP* followed by *BGN, MMP2* and *FBN1* showed the highest levels of expression, whereas the lowest levels of expression were observed for *RUNX2* and *SPP1* and *ANKH* in some adult samples (Table 4).

In the aortic arch many of the selected genes were not significantly changed through development (Table 4 and Supplementary Figure S6) *FBN2* expression decreased in 2-year old sheep compared to newborn and 1 week old lambs (*p* < 0.01). *SPP1* expression was also significantly lower in adult sheep compared to newborn and 1 week old lambs (*p* < 0.05). In contrast the mineralization inhibitor genes *TNFRSF11B* and *MGP* increased with age as did *RUNX2.* Within the aortic arch, the highest levels of expression were seen in *MGP, BGN* and *ENPP1*, and the lowest in *RUNX2* and *TNFRSF11B* (newborn lambs) and *FBN2* and *SPP1* (2-year old adults) (Table 4).

In the abdominal aorta, expression of the mRNAs of ECM protein-encoding genes *COL1A1* and *FBN1* peaked in 8-week old lambs (Supplementary Figure S7) while *FBN2* expression decreased from 8 weeks of age to 2 years of age (*p* < 0.01). *TIMP1* expression was found to increase with age. For the key calcification genes, *SPP1* showed a reduction in its expression from 8-week old lambs to 2-year old sheep (*p* < 0.05), and *RUNX2* was decreased from newborn to 8-week old lambs (*p* < 0.05; Supplementary Figure S7). *ANKH* and *TNFRSF11B* expression was significantly increased in the 8-week old and 2-year old sheep compared to newborn lambs (*p* < 0.05). In the abdominal aorta, the highest levels of expression were observed for *MMP2, MGP, BGN* and *FBN1*, and the lowest overall for *RUNX2* (Table 4).

### Vascular Calcification (VC) Inhibitors Expressed in the Healthy Adult Cardiovascular System

Using RT-qPCR, the expression profiles of various key inhibitors of ectopic calcification in the cardiovascular system (*ANKH, ENPP1, FBN1, MGP, NT5E, TNFRSF11B*) were investigated in different cardiovascular regions, in the six adult sheep. Figure 3 summarizes where these genes were highly expressed in the cardiovascular tissues. Overall, the key VC genes tended to be more highly expressed in the cardiac valves than the other tissues; expression in the myocardium was lowest (Figure 3).

**FIGURE 4:**
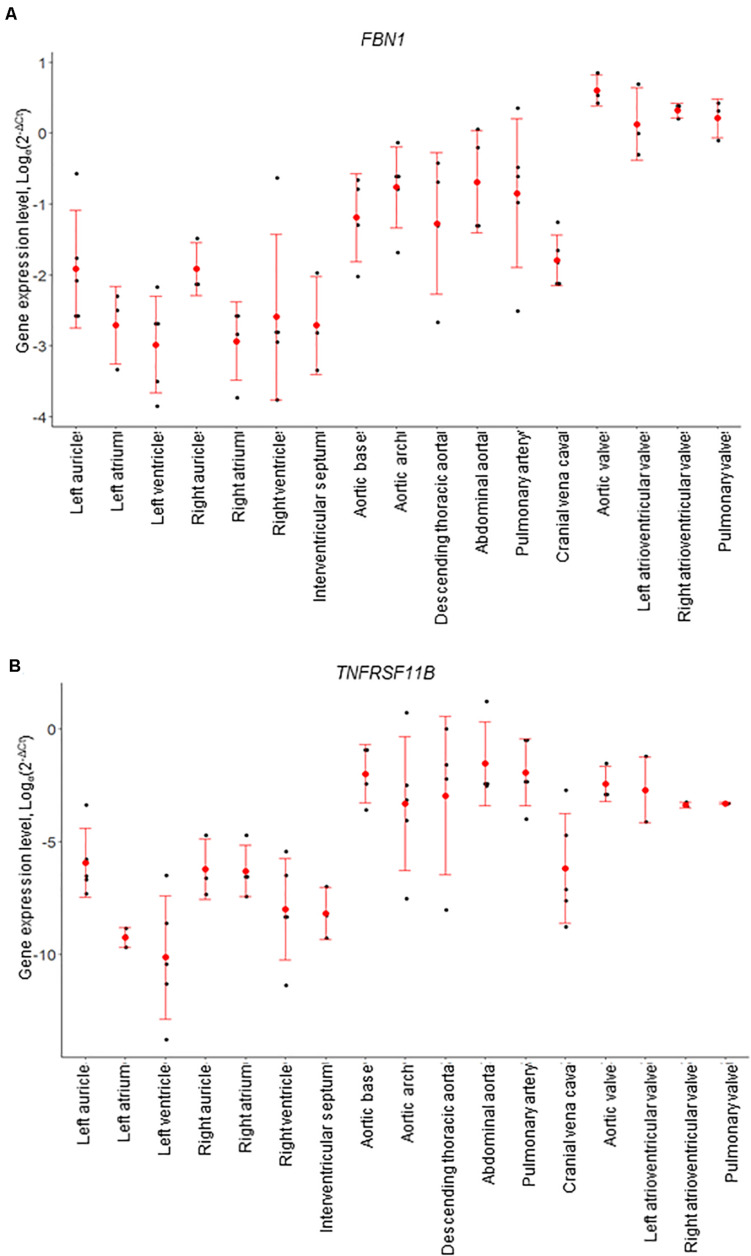
mRNA expression levels for individual animals, determined by RT-qPCR. **(A)** *FBN1* and **(B)** *TNFRSF11B* (osteoprotegerin). Gene expression levels were normalized to the geomean of *GAPDH* and *YWHAZ*. Dot plots show individual data points (black dot), the mean expression for each tissue (red dot) and standard deviation (red error bars).

Of the genes examined, *FBN1* and *TNFRSF11B* showed the greatest variation across the cardiovascular system. *FBN1* was most highly expressed in the valves, which had significantly higher expression than the myocardium and vena cava tissues (*p* < 0.01; Figure 4). Overall, *FBN1* expression was approximately 4-fold lower in the aortic samples than the cardiac valves (*p* < 0.05), There was no significant difference between the aortic arch compared to the left AV valve, the abdominal aorta compared to the left AV, right AV and pulmonary valves, and the pulmonary artery compared to the left AV valve (Figure 4). Expression of *FBN1* in the arteries was in general higher (4-fold) than in the myocardium and cranial vena cava (*p* < 0.05). *TNFRSF11B* expression was higher in the arteries and cardiac valves compared to the myocardium and cranial vena cava (Figure 4). The levels of *TNFRSF11B* expression in the aortic samples, pulmonary artery, aortic valve and left AV valve were significantly higher compared to the myocardium and cranial vena cava (*p* < 0.05; Figure 4). The expression of *TNFRSF11B* was generally very low in the myocardium (approximately 1000-fold lower than in the arteries) (Figure 4).

Of the other vascular calcification genes investigated, *MGP* showed the highest levels of expression compared to the other tested genes with expression being similar in all tissues, although the aortic valve showed higher expression than the aortic arch, the cranial vena cava and the myocardial tissues (*p* < 0.05; Supplementary Figure S8). The expression of *ANKH* was similar in all tested tissues, but the pulmonary artery showed significantly higher expression levels than the right atrium and left auricle (*p* < 0.05; Supplementary Figure S8). *NT5E* expression was found to be higher in the cardiac valves and the pulmonary artery compared to the other tissues included in this study (*p* < 0.05; Supplementary Figure S9). Some arterial tissues exhibited higher expression than the myocardial samples, including the aortic root, aortic arch and abdominal aorta compared to the right atrium, left ventricle and interventricular septum (*p* < 0.05; Supplementary Figure S9). The expression of *ENPP1* was significantly higher in the cardiac valves and aortic arch compared to the myocardium and vena cava (*p* < 0.05; Supplementary Figure S10). The remaining aortic samples showed intermediate expression between the valves and myocardium (Supplementary Figure S10). Similarly the expression of *SPP1* was found to be higher in the cardiac valves compared to the myocardium and the aortic root (*p* < 0.05; Supplementary Figure S10). Other than in the cardiac valves, *SPP1* expression was generally very low in the tested cardiovascular tissues, reaching levels similar to that of the bone marker *RUNX2* (Supplementary Figure S10). The expression of *RUNX2* was low in all tested samples. However, the aortic valve showed higher *RUNX2* expression compared to the myocardium, the cranial vena cava and aortic root (*p* < 0.05; Supplementary Figure S9).

### Comparative Analysis of RT-qPCR and RNA-seq Results

We examined the genes analyzed by qPCR in the network analysis. The cluster and expression level (as TPM) for each of these genes are included in Supplementary Dataset S2. In summary, many of the genes analyzed by RT-qPCR were included in the large cluster 1 (high in cardiac valves, lower in muscle). This included *COL1A1, FBN2, MMP2, TIMP1, COL3A1*. Others were in Clusters 22 and 24, which showed a bigger differential between the valves (high) and the muscle (low), including *ADAMTS6, SMAD2, NFIL3, FMOD, FBN1*. Cluster 3, in which the aortic valve showed higher expression than the other cardiac valves, and all valves were higher than the muscle samples, contained *COL1A2, BGLAP, BGN*. Two genes of interest (*NPPA* and *DKK3*) were in Cluster 36 (high only in auricles). Some of the genes of interest were not detected in the network analysis (e.g., *RUNX2* and *ANKH*), either because their expression level in the healthy tissues was too low or they did not correlate with any other gene at the correlation coefficient of 0.99 used.

## Discussion

The maintenance of a healthy cardiovascular system requires expression of genes that contribute to essential biological activities and repression of those that are associated with functions likely to be detrimental to cardiovascular homeostasis. As cardiovascular disease is of major clinical importance, understanding the roles of genes in co-expression networks and their associated molecular pathways will be useful in understanding their dysregulation in pathological events. Detailed analysis of gene expression of tissues and cell types in the cardiovascular system provides a powerful resource for investigation of healthy cardiovascular system function (reviewed in Wirka et al., 2018). However, analysis of the cardiovascular system in humans is often jeopardized due to tissues being only available post-mortem, where frequently the health status of the individual is not known and the quality of the RNA may be poor (Ferreira et al., 2018). A large animal model, where tissues can be collected quickly post mortem from healthy animals, offers the opportunity to perform a detailed characterization of the mammalian cardiovascular transcriptome. In this study we took advantage of the recently published sheep gene expression atlas (Clark et al., 2017; BioGPS, 2020) to examine individual components of the cardiovascular system in the sheep, which are similar to humans in their physiology and genetics (reviewed in Hamernik, 2019). We then explored a number of genes related to ectopic calcification and the extracellular matrix in additional tissues and developmental stages, from the same animals, but not initially presented as part of the sheep transcriptional atlas, using RT-qPCR. The insights from this novel analysis of the sheep cardiovascular system will be valuable in understanding the physiology of the healthy mammalian cardiovascular system and will help to facilitate the development of clinical and therapeutic approaches for the prevention and treatment of cardiovascular diseases, particularly those related to ectopic calcification.

Our results from both approaches demonstrate that there is extensive expression of genes encoding proteins involved in formation and maintenance of the extracellular matrix (ECM) in cardiovascular tissues, particularly the cardiac valves and aorta. The cardiovascular system consists of specialized cells surrounded by a dynamic ECM that not only provides structure through connections of cells within the network, but also directs cellular function. The ECM is an important provider of structural and biomechanical support, and helps to regulate molecular interactions between growth factors and cell surface receptors (Kim et al., 2011; Davis and Summers, 2012). The ECM is also necessary for providing mechanical signals that result in cell responses including migration, proliferation and apoptosis. A detailed understanding of the gene expression profile underpinning how cells respond to the ECM by remodeling their microenvironment in the healthy cardiovascular system is crucial, given the dysregulation of this remodeling process in cardiovascular-related diseases including ectopic calcification, atherosclerosis and hypertension (Ponticos and Smith, 2014). The cardiac valves showed higher expression of a range of ECM genes relative to cardiac muscle, both by RNA-seq and by RT-qPCR. This is consistent with continuous remodeling of the ECM in cardiac valves due to the normal functional stresses (mechanical and blood-flow induced shear) that the valve is subjected to. During development, most ECM genes decreased in expression, particularly in the left ventricle, intraventricular septum and aortic root. This accords with the results from RNA-seq, where the heart muscle from adult sheep showed lower expression of ECM genes than the cardiac valves, and probably reflects the completion of organ development, after which the requirement for expression would depend on the turnover of the proteins to maintain ECM homeostasis. However, with aging, expression and *de novo* synthesis may not be sufficient to balance turnover of the proteins, leading to a loss of structural support in the ECM over time. Different ECM genes were activated at different times during development. For example, two members of the fibrillin family, key components of the ECM (Sakai et al., 1986; Ramirez and Pereira, 1999; Davis and Summers, 2012) were expressed in the cardiovascular system. The gene encoding FBN2, traditionally regarded as a fetal protein which is involved at the beginning of elastogenesis and early morphogenesis (Zhang et al., 1995), appeared to be activated earlier in cardiovascular development than the gene for FBN1, which has been attributed functions late in morphogenesis and organogenesis (Zhang et al., 1995). Both genes exhibited highest expression in the cardiac valves and decreased in expression during cardiovascular development. Expression of *FBN1* mRNA in the aorta supports the role of FBN1 in maintaining the structural integrity of this major artery. In Marfan syndrome, the dysfunction of FBN1 leads to aortic aneurysms and elastic fiber calcification (Pereira et al., 1999; Bunton et al., 2001). Both RNA-seq and RT-qPCR results showed highest expression of *FBN1* in the cardiac valves, particularly the aortic valve, consistent with the valve failure seen in patients with Marfan syndrome. Other ECM genes that decreased with age included *COL1A1* and *BGN.* The ECM proteases known as matrix metallopeptidases (MMPs) and their tissue inhibitors, TIMPs, are important modulators of matrix protein turnover (Elmore et al., 1998; Hughes and Jacobs, 2017). It is thought that alterations of the balance between MMPs and TIMPs are critical in the formation of aortic aneurysms and age-associated physiological changes in the cardiovascular system (Rabkin, 2014; Meschiari et al., 2017). In this study, the level of *MMP2* expression was amongst the highest of the genes examined in the abdominal aorta, and *TIMP1* expression was found to increase with age in this tissue. The increase in *TIMP1* expression may help prevent the development of abdominal aortic aneurysms in a healthy animal by inhibiting degradation of ECM structural proteins. MMPs and TIMPs may also be crucial in myocardial function, where increases in their levels have been found to correlate with age in human and mouse (Meschiari et al., 2017). Furthermore, these ECM regulators have also been reported to be important in the remodeling process in the left ventricle after experimentally induced myocardial infarction in mice, where the local endogenous control of MMPs by TIMP1 was suggested to be important for the ECM structure, as well as myocardial function and myocyte growth (Creemers et al., 2003). In the RNA-seq analysis, both *MMP2* and *TIMP1* mRNAs were high in the cardiac valves (both at around 600 TPM) and minimal in the heart and skeletal muscle, suggesting that they balance each other in healthy tissue. Additional studies on the expression of other MMPs and TIMPs may be useful to determine their involvement in the development of CVD.

We detected transcripts associated with macrophages in all samples in the RNA-seq analysis, notably enriched in the valves. The homeostatic functions of resident macrophages in arterial and cardiac tissue have been widely studied (Swirski et al., 2016; Lim et al., 2018). The presence of resident macrophages in human and mouse valve tissue has also been recognized previously (Sraeyes et al., 2018). In the mouse, heterogeneous resident valve macrophage populations are established in the postnatal period and the population is expanded by monocyte recruitment in a model of myxomatous disease (Hulin et al., 2018). Damaged cardiac valves are prone to life-threatening infectious and non-infectious endocarditis (Yang and Frazee, 2018), which is common in elderly humans, and ongoing surveillance and repair are necessary to prevent pathological outcomes. The sheep is an ideal animal to investigate the aging heart further, since the sheep life span is around 10 years^11^ and elderly animals can be obtained from commercial sources at the end their productive life, rather than needing to be aged for the investigation.

Cardiovascular calcification is a common occurrence in patients affected with numerous devastating chronic diseases including diabetes, chronic kidney disease (CKD), and atherosclerosis. It is also a hallmark of rare genetic diseases including pseudoxanthoma elasticum (PXE), generalized arterial calcification of infancy (GACI), Keutel syndrome, and progeria (Rashdan et al., 2016). Endogenous calcification inhibitors represent a crucial defense mechanism against cardiovascular calcification, as recently highlighted by a consensus statement from the COST Action EuroSoftCalcNet (Bäck et al., 2019). A striking finding of our analysis was the expression of genes associated with bone formation and ectopic calcification in the cardiovascular system of healthy sheep throughout development. These included both genes encoding proteins that promote bone formation and calcification (such as *SPP1, SPARC, BMP4* and *BGLAP*) and those which suppress mineralization (such as *ENPP1, ANKH, FBN1, MGP, TNFRSF11B* and *NT5E*). Ectopic calcification can develop in various tissues, and many reports include the aorta and the aortic valve as sites of VC (Demer and Tintut, 2009; New and Aikawa, 2011). The expression of genes associated with suppression of bone formation would likely be advantageous in preventing VC, but the predisposing factors and pathways that infer the susceptibility of specific tissues to calcification are still unknown. Moreover, differences in the mechanisms behind intimal, median and valvular calcification may exist (Côté et al., 2012; Patel et al., 2017; Qian et al., 2017). Expression of *ENPP1* decreased throughout development. ENPP1 has a role in regulating extracellular nucleotide levels and potentially a dual role in VC (Côté et al., 2012). ENPP1 may contribute to normal cardiovascular function through the regulation of extracellular ATP concentrations and the generation of the calcification inhibitor PPi (Nam et al., 2011; Côté et al., 2012). Deficiency of *ENPP1* leads to generalized arterial calcification (Mackenzie et al., 2012). *ANKH* mRNA was also increased. ANKH transports cytoplasmic PPi out of the cell (Harmey et al., 2004). ANKH may provide a protective effect against the development of VC, since patients with VC have been found to have decreased *ANKH* expression (Zhao et al., 2012). MGP is also a calcification inhibitor, possibly via its ability to block BMP signaling (Zebboudj et al., 2002; Yao et al., 2010). RNA-seq analysis showed that *SPP1* was highest in the aortic and left atrioventricular valves (Cluster 57) and *SPARC, BMP4*, and *BGLAP* were all in Cluster 3, also with highest expression in aortic valves and slightly lower in the other valves, consistent with a role in promoting mineralization in these sites. Calcification inhibitors *ENPP1, FBN1, MGP, TNFRSF11B*, and *NT5E* were in different clusters, all with high expression in the valves. *MGP* expression was extremely high (∼32,000 TPM) in aortic valve, possibly to balance the expression of *SPARC* (also high at 1,800 TPM). Expression of *BMP4* and *SPARC* in the cardiac valves in the sheep gene expression atlas dataset analyzed here, supports the importance of calcification inhibitors like MGP in preventing the development of calcification, especially in tissues which express genes associated with bone development. The expression of *MGP* was consistently high in all the different ages and tissues investigated and this factor may play a cardioprotective role against the development of calcification. *SPP1* encodes secreted phosphoprotein 1, also known as osteopontin, which is associated with bone formation and calcification, and is a constituent of normal elastic fibers in the aorta and skin (Rutsch et al., 2011). SPP1 in the valves is likely to be associated with the resident macrophages, since it was the most highly expressed transcript in isolated macrophages in the sheep atlas, at least 100-fold higher than in any tissue other than placenta (Clark et al., 2017; BioGPS, 2020). *SPP1* is similarly macrophage-enriched in humans [Fantom Consortium and the Riken PMI and CLST (DGT), 2014; Lizio et al., 2015] and pig (Summers et al., 2020). Increased *SPP1* mRNA expression and plasma osteopontin levels have been linked with cardiac allograft vascular disease (CAVD) (Rajamannan et al., 2003; Yu et al., 2009), whereas it has been reported to have inhibitory effects on arterial calcification (Wada et al., 1999; Speer et al., 2002). Examples of its reported roles include bone remodeling, anti-apoptotic signaling and inflammatory regulation (Denhardt et al., 2001). SPP1 can exist in different states (phosphorylated and glycosylated), and it is thought that these specific forms have distinct functions (Denhardt et al., 2001). There was a decrease in expression of *SPP1* mRNA with age in the sheep cardiovascular tissues. Increased expression of *SPP1* has been implicated in VC development, as well as coronary artery disease and heart failure (Rosenberg et al., 2008; New and Aikawa, 2011; Dai et al., 2014). Our results suggest that SPP1 is important in the earlier stages of cardiovascular development, whereas higher expression in later life may lead to these adverse clinical outcomes. *ENPP1* was also strongly macrophage-enriched in the wider sheep atlas (Clark et al., 2017; BioGPS, 2020). The expression profiles of *SPP1* and *ENPP1* were very similar suggesting that they contribute to a balance between promotion and suppression of calcification in cardiovascular tissues. *MGP* expression was high compared to the other genes in this study. Although it has been established that *MGP* has a role in the inhibition of VC, its particular role within the cardiovascular system is still unclear. As with SPP1, MGP can exist in different states, and the levels of these different states are thought to affect the CVD risk of an individual (Dalmeijer et al., 2013). Elevated dephosphorylated MGP (dpMGP) has been found in patients with CKD, heart failure, CAVD, aortic stenosis and other CVD events (Schurgers et al., 2008; Mayer et al., 2014; Vassalle and Iervasi, 2014). The locally produced active form of MGP (phosphorylated and gammacarboxylated) has been implicated to have cardioprotective effects (Schurgers et al., 2010; El Asmar et al., 2014; Liu et al., 2015) such as through its inhibition of VC, where it has been reported to inhibit BMP signaling (Zebboudj et al., 2002; Yao et al., 2010). In addition, decreased active MGP was found in aortic valvular interstitial cells (VICs) derived from patients with CAVD (Venardos et al., 2015). More studies into the numerous genes that have been implicated in VC and into the post-translational processing the proteins undergo are required in order to understand their expression patterns within the cardiovascular system, and to gain additional insights into their physiological functions.

One outcome of this study is the functional annotation of previously novel genes. At present, there are many predicted mammalian protein-coding loci and non-protein-coding genes that are yet to have informative annotation (Oliver, 2000; Klomp and Furge, 2012). Protein-coding genes that contribute to common generic and cell-specific cellular processes or pathways generally form co-expression clusters, allowing the inference of the function of a gene (of previously unknown function) using the ‘guilt-by-association’ principle (Oliver, 2000; Freeman et al., 2012; Klomp and Furge, 2012). Martherus et al. (2010), for example, used this method effectively to identify heart enriched mitochondrial genes. We have previously identified novel macrophage and nervous system specific genes (Hume et al., 2010; Carpanini et al., 2017). In the pig we found that many unannotated transcripts were fragments of known genes; these were highlighted because they shared expression pattern with the annotated fragment of the same gene (Summers et al., 2020). In our study a number of co-expression clusters were found that distinguished the cardiac valves from heart muscle. The novel (unannotated) genes within the tissue-specific clusters described here potentially have the same functions as other genes in the cluster, which allows for functional annotation of these genes. For example, the gene *ENSOARG00000005484* from Cluster 36 encodes a protein involved in calcium ion transport across membranes, consistent with the other ion channel genes in this cluster. The high level of expression of some of these novel genes suggests that they are an important part of the process of development and differentiation in the cardiovascular system. As such they warrant further investigation using knock out animals or functional validation in relevant cell lines using CRISPR to examine consequences of their dysfunction (as reviewed in van Kampen and van Rooij, 2019).

As the RNA-seq analysis we present here only included seven cardiovascular tissues (three cardiac valves and the four chambers of the heart), we were not able to define gene expression clusters associated with other cardiovascular tissues, such as the veins, arteries and other regions of the heart. We used RT-qPCR to examine a limited number of genes in the extended cardiovascular system at several developmental stages. Transcriptomic analysis using RNA-seq of a wider sub-set of samples, including more tissue types and developmental stages, would identify specific expression patterns, for example for different parts of the aorta. In addition, we did not cluster the cardiovascular samples with other tissues (other than a representative of skeletal muscle) from the wider sheep gene expression atlas dataset (Clark et al., 2017; BioGPS, 2020) since this study focused on transcriptional differences within the sheep cardiovascular system. The results should be validated using functional analysis of the encoded proteins as mRNA levels do not necessarily reflect protein levels, and many proteins (such as MGP discussed above) must be modified post-translationally for full activity. The study is also limited by the lack of fetal arterial tissues. Earlier studies in mouse and human (Aikawa et al., 2006; Cui et al., 2019; Hulin et al., 2019) have shown transcriptomic differences in human cardiac valves before and after birth with changes in collagen and elastin content and structure and differences in activation of vascular endothelial and interstitial cells with the switch from fetal to neonatal circulation. It would be interesting to know whether the same is true for the developing arteries.

In summary we have used RNA-seq and RT-qPCR results from the sheep heart and cardiac valves to further explore the transcriptome of the cardiovascular system in this large animal. These data provide initial insights into tissue-specific expression of key genes, which will be useful in understanding their physiological function in a healthy mammal. This study will support future research into the functions of implicated genes in the development of VC, and increase the utility of the sheep as a model in cardiovascular research. The analysis of further tissues and developmental stages, such as a wider range of prenatal ages and elderly animals would provide further insight into the gene expression patterns of key genes implicated in the progression of important cardiovascular functions or disease with age, and is feasible using the sheep as a model. Here we have built a foundation to explore the transcriptome of the developing and aging cardiovascular system and provided a highly useful comprehensive resource. Recent advances in single cell RNA-seq technology provide a new frontier to understand cell type specific gene expression and will allow us to further de-convolute expression patterns in cardiovascular tissues (Chaudhry et al., 2019). Further in-depth studies will be necessary to understand the gene expression networks and molecular pathways that exist in the different cardiovascular structures, and how they develop and change as the cardiovascular system matures.

## Data Availability Statement

The raw RNA-seq data have been deposited in the European Nucleotide Archive (ENA) under study accession number PRJEB19199. The dataset of gene expression estimates as TPM from Kallisto is available through the Edinburgh Datashare Portal at https://doi.org/10.7488/ds/2808.

## Ethics Statement

The animal study was reviewed and approved by The Roslin Institute’s Animal Welfare and Ethical Review Body (AWERB).

## Author Contributions

DH acquired the funding for the sheep gene expression atlas. EC coordinated and designed the sheep gene expression atlas with assistance from DH and KS. H-GT, EC, and KS performed the sample collection from sheep. H-GT and EC performed the RNA extractions. SB performed the all bioinformatic analyses. H-GT, GM, and KS performed the network cluster analysis. H-GT performed the RT-qPCR analysis. H-GT interpreted the results with VM, BC, and KS. H-GT wrote the manuscript with GM, EC, KS, and VM. All authors contributed to the article and approved the submitted version.

## Conflict of Interest

The authors declare that the research was conducted in the absence of any commercial or financial relationships that could be construed as a potential conflict of interest.

## Footnotes

1 http://biogps.org/

2 https://www.ebi.ac.uk/gxa

3 http://www.ebi.ac.uk/ena/data/view/PRJEB19199

4 http://biogps.org/dataset/BDS_00015/sheep-atlas/

5 http://biolayout.org/

6 https://david.ncifcrf.gov/

7 https://www.ensembl.org/Ovis_aries/Info/Annotation

8 http://primer3.ut.ee/

9 https://www.r-project.org/

10 https://www.biogps.org/sheepatlas

11 http://www.sheep101.info

## Supplementary Material

FIGURE S1 RNA-seq expression profiles of selected genes. Expression levels were measured using RNA-seq and shown as median expression levels in transcripts per million (TPM; *n* = 4–6). *Y* axis shows normalized median TPM (Bush et al., 2017). **(A–D)** Cluster 1 gene expression profiles. Genes include *COL1A1* (collagen type I alpha 1), *COL3A1* (collagen type III alpha 1), *MMP*2 (matrix metalloproteinase 2) and *TIMP1* (tissue inhibitor of metalloproteinases 1). **(E–G)** Cluster 3 gene expression profiles. Genes include *COL1A2* (collagen type I alpha 2), *BGLAP* (bone gamma-carboxyglutamate protein) and *BGN* (biglycan). **(H–J)** Cluster 22 gene expression profiles. Genes include *ENPP1* (ectonucleotide pyrophosphate/phosphodiesterase 1), *ADAMTS6* (ADAM metallopeptidase with thrombospondin type 1 motif 6) and *SMAD2* (SMAD family member 2). **(K,L)** Cluster 24 gene expression profiles. Genes include *FBN1* (fibrillin 1) and *FMOD* (fibromodullin). **(M–O)** Cluster 36 gene expression profiles. Genes include *NPPA* (natriuretic peptide A) and *DKK3* (Dickkopf WNT signaling pathway inhibitor 3).

FIGURE S2 Gene expression profiles during development in the left ventricle. Genes include: **(A)** collagen type I alpha 1, *COL1A1*, **(B)** biglycan, *BGN*, **(C)** matrix metalloproteinase 2, *MMP2*, **(D)** TIMP metallopeptidase inhibitor 1, *TIMP1*, **(E)** fibrillin 1, *FBN1*, **(F)** fibrillin 2, *FBN2*, **(G)** secreted phosphoprotein1/osteopontin, *SPP1*, **(H)** progressive ankylosis protein, *ANKH*, **(I)** osteoprotegerin, *TNFRSF11B*, and **(J)** Runt-related transcription factor 2, *RUNX2*. Black dots show gene expression from individual animals (*n* = 3–5) and red dot and error bars show the mean ± standard deviation (SD) per tissue. Gene expression levels were normalized to the geomean of *GAPDH* and *YWHAZ*. In blue, asterisk (^∗^) denotes significant differences compared to fetal d100 sheep, triangle (Δ) compared to newborn sheep and circle (∘) compared to 8 week old sheep, where 1 symbol = 0.01 < *p* < 0.05, 2 symbols = 0.001, *p* < 0.01 and 3 symbols = *p* < 0.001.

FIGURE S3 Gene expression profiles during development in the interventricular septum. Genes include: **(A)** collagen type I alpha 1, *COL1A1*, **(B)** biglycan, *BGN*, **(C)** matrix metalloproteinase 2, *MMP2*, **(D)** fibrillin 1, *FBN1*, **(E)** fibrillin 2, *FBN2*, **(F)** secreted phosphoprotein1/osteopontin, *SPP1*, **(G)** progressive ankylosis protein, *ANKH*, and **(H)** matrix Gla protein, *MGP*. Black dots show gene expression from individual animals (*n* = 3–5) and red dot and error bars show the mean ± standard deviation (SD) per tissue. Gene expression levels were normalized to the geomean of *GAPDH* and *YWHAZ*. In blue, asterisk (^∗^) denotes significant differences compared to fetal d100 sheep, triangle (Δ) compared to newborn sheep, circle (o) compared to 1 week old sheep and square (□) compared to 8 week old sheep, where 1 symbol = 0.01 < *p* < 0.05, 2 symbols = 0.001 < *p* < 0.01 and 3 symbols = *p* < 0.001.

FIGURE S4 Gene expression profiles during development in the pulmonary artery. Genes include: **(A)** TIMP metallopeptidase inhibitor 1, *TIMP1*, **(B)** fibrillin 2, *FBN2*, **(C)** ectonucleotide pyrophosphatase/phosphodiesterase 1, *ENPP1*, **(D)** secreted phosphoprotein1/osteopontin, *SPP1*, and **(E)** Runt-related transcription factor 2, *RUNX2*. Black dots show gene expression from individual animals (*n* = 3–5) and red dot and error bars show the mean ± standard deviation (SD) per tissue. Gene expression levels were normalized to the geomean of *GAPDH* and *YWHAZ*. In blue, asterisk (^∗^) denotes significant differences compared to newborn sheep, triangle (Δ) compared to 8 week old sheep, where 1 symbol = 0.01 < *p* < 0.05, 2 symbols = 0.001 < *p* < 0.01 and 3 symbols = *p* < 0.001.

FIGURE S5 Gene expression profiles during development in the aortic root. Genes include: **(A)** collagen type I alpha 1, *COL1A1*, **(B)** biglycan, *BGN*, **(C)** matrix metalloproteinase 2, *MMP2*, **(D)** fibrillin 1, *FBN1*, **(E)** fibrillin 2, *FBN2*, **(F)** ectonucleotide pyrophosphatase/phosphodiesterase 1, *ENPP1*, and **(G)** secreted phosphoprotein1/osteopontin, *SPP1*. Black dots show gene expression from individual animals (*n* = 3–5) and red dot and error bars show the mean ± standard deviation (SD) per tissue. Gene expression levels were normalized to the geomean of *GAPDH* and *YWHAZ*. In blue, asterisk (^∗^) denotes significant differences compared to newborn sheep, triangle (Δ) compared to 1 week old sheep and circle (o) compared to 8 week old sheep, where 1 symbol = 0.01 < *p* < 0.05, 2 symbols = 0.001 < *p* < 0.01 and 3 symbols = *p* < 0.001.

FIGURE S6 Gene expression profiles during development in the aortic arch. Genes include: **(A)** fibrillin 2, *FBN2*, **(B)** secreted phosphoprotein1/osteopontin, *SPP1*, **(C)** matrix Gla protein, *MGP*, **(D)** osteoprotegerin, *TNFRSF11B*, and **(E)** Runt-related transcription factor 2, *RUNX2*. Black dots show gene expression from individual animals (*n* = 3–5) and red dot and error bars show the mean ± standard deviation (SD) per tissue. Gene expression levels were normalized to the geomean of *GAPDH* and *YWHAZ*. In blue, asterisk (^∗^) denotes significant differences compared to newborn sheep and triangle (Δ) compared to 1 week old sheep, where 1 symbol = 0.01 < *p* < 0.05, 2 symbols = 0.001 < *p* < 0.01 and 3 symbols = *p* < 0.001.

FIGURE S7 Gene expression profiles during development in the abdominal aorta. Genes include: **(A)** collagen type I alpha 1, *COL1A1*, **(B)** TIMP metallopeptidase inhibitor 1, *TIMP1*, **(C)** fibrillin 1, *FBN1*, **(D)** fibrillin 2, *FBN2*, **(E)** secreted phosphoprotein1/osteopontin, *SPP1*, **(F)** progressive ankylosis protein, *ANKH*, **(G)** osteoprotegerin, *TNFRSF11B*, and **(H)** Runt-related transcription factor 2, *RUNX2*. Black dots show gene expression from individual animals (*n* = 3–5) and red dot and error bars show the mean ± standard deviation (SD) per tissue. Gene expression levels were normalized to the geomean of *GAPDH* and *YWHAZ*. In blue, asterisk (^∗^) denotes significant differences compared to newborn sheep and triangle (Δ) compared to 8 week old sheep, where 1 symbol = 0.01 < *p* < 0.05, 2 symbols = 0.001 < *p* < 0.01 and 3 symbols = *p* < 0.001.

FIGURE S8 mRNA expression profile for **(A)** matrix Gla protein (*MGP*), **(B)** progressive ankylosis protein homolog (*ANKH*). Gene expression levels were normalized to the geomean of *GAPDH* and *YWHAZ*. Dot plots show individual data points (black dot), the mean expression for each tissue (red dot) and standard deviation (red error bars).

FIGURE S9 mRNA expression profile for **(A)** ecto-5′-nucleotidase (*NT5E*) and **(B)** Runt-related transcription factor 2 (*RUNX2*). Gene expression levels were normalized to the geomean of *GAPDH* and *YWHAZ*. Dot plots show individual data points (black dot), the mean expression for each tissue (red dot) and standard deviation (red error bars).

FIGURE S10 mRNA expression profile for **(A)** ectonucleotide pyrophosphatase/phosphodiesterase 1 (*ENPP1*) and **(B)** secreted phosphoprotein1/osteopontin (*SPP1*). Gene expression levels were normalized to the geomean of *GAPDH* and *YWHAZ*. Dot plots show individual data points (black dot), the mean expression for each tissue (red dot) and standard deviation (red error bars).

TABLE S1 Details of tissues sequenced to generate the RNA-seq dataset for the cardiovascular gene expression atlas. Skeletal muscle (bicep) was also included, as an example of skeletal muscle tissue, for comparative analysis. All libraries were Illumina 125 bp paired end stranded libraries.

TABLE S2 Minimally annotated genes in clusters with defined expression patterns.

TABLE S3 Genes examined in sheep cardiovascular tissues during development.

DATASET S1 Gene expression estimates as transcripts per million (TPM) for seven cardiovascular tissues and skeletal muscle (bicep) generated for the sheep gene expression atlas using Kallisto.

DATASET S2 Details of the genes in the clusters and average expression profiles for each cluster from the gene to gene network analysis presented in Figures 1, 2. Pearson correlation co-efficient *r* ≥ 0.99, MCL (inflation = 2.2).

DATASET S3 RT-qPCR results for all genes analyzed including standard deviations.
