# Supplementary material for: Expression of Calcification and Extracellular Matrix Genes in the Cardiovascular System of the Healthy Domestic Sheep (Ovis aries)
Source: Front Genet. 2020 Sep 8;11:919. doi: 10.3389/fgene.2020.00919 (PMC7506100; doi:10.3389/fgene.2020.00919)
Supplement: FIGURE S1 — RNA-seq expression profiles of selected genes. Expression levels were measured using RNA-seq and shown as median expression levels in transcripts per million (TPM; n = 4–6). Y axis shows normalized median TPM (Bush et al., 2017). (A–D) Cluster 1 gene expression profiles. Genes include COL1A1 (collagen type I alpha 1), COL3A1 (collagen type III alpha 1), MMP2 (matrix metalloproteinase 2) and TIMP1 (tissue inhibitor of metalloproteinases 1). (E–G) Cluster 3 gene expression profiles. Genes include COL1A2 (collagen type I alpha 2), BGLAP (bone gamma-carboxyglutamate protein) and BGN (biglycan). (H–J) Cluster 22 gene expression profiles. Genes include ENPP1 (ectonucleotide pyrophosphate/phosphodiesterase 1), ADAMTS6 (ADAM metallopeptidase with thrombospondin type 1 motif 6) and SMAD2 (SMAD family member 2). (K,L) Cluster 24 gene expression profiles. Genes include FBN1 (fibrillin 1) and FMOD (fibromodullin). (M–O) Cluster 36 gene expression profiles. Genes include NPPA (natriuretic peptide A) and DKK3 (Dickkopf WNT signaling pathway inhibitor 3). [file Data_Sheet_1.PDF]

Expression of calcification and extracellular matrix genes in the cardiovascular system of the healthy domestic sheep (*Ovis aries*).

Hiu-Gwen Tsang[1v], Emily L. Clark[1v], Greg R. Markby[1], Stephen J. Bush[1,2], David A. Hume[3], Brendan M. Corcoran[4], Vicky E. MacRae[1*] and Kim M. Summers[1,3*]

[1]The Roslin Institute and R(D)SVS, The University of Edinburgh, Easter Bush, EH25 9RG, UK.

[2]Nuffield Department of Clinical Medicine, John Radcliffe Hospital, University of Oxford, Oxford, UK

[3]Mater Research Institute-University of Queensland, Translational Research Institute, Woolloongabba QLD 4102 Australia

[4]Royal (Dick) School of Veterinary Studies, University of Edinburgh, Easter Bush, EH25 9RG, UK

Supplementary Tables

Supplementary Table 1. Details of tissues sequenced to generate the RNA-seq dataset for the cardiovascular gene expression atlas.
Supplementary Table 2. Minimally annotated genes in clusters with defined expression patterns.
Supplementary Table 3. diseases associated with genes examined in sheep cardiovascular tissues during development.

Supplementary Figures

Supplementary Figure 1. RNA-seq expression profiles of selected genes.
Supplementary Figure 2. Gene expression profiles during development in the left ventricle**.**
Supplementary Figure 3. Gene expression profiles during development in the interventricular septum**.**
Supplementary Figure 4. Gene expression profiles during development in the pulmonary artery.
Supplementary Figure 5. Gene expression profiles during development in the aortic root.
Supplementary Figure 6. Gene expression profiles during development in the aortic arch.
Supplementary Figure 7. Gene expression profiles during development in the abdominal aorta.
Supplementary Figure 8. mRNA expression profile for (A) matrix Gla protein (*MGP*) and (B) progressive ankylosis protein homologue (*ANKH*).
Supplementary Figure 9. mRNA expression profile for (A) ecto-5’-nucleotidase (*NT5E*) and (B) Runt-related transcription factor 2 (*RUNX2*).
Supplementary Figure 10. mRNA expression profile for (A) ectonucleotide pyrophosphatase/phosphodiesterase 1 (*ENPP1*) and (B) secreted phosphoprotein 1/osteopontin (*SPP1*).

Tissue	Library Type	Sequencing Depth	Number of Individuals
Left ventricle	Total RNA-seq	>100 million reads per sample	3 male, 3 female
Aortic valve	mRNA-seq	>25 million reads per sample	2 male, 2 female
Left atrioventricular (Mitral) valve	mRNA-seq	>25 million reads per sample	2 male, 2 female
Right atrioventricular (Tricuspid) valve	mRNA-seq	>25 million reads per sample	3 male, 1 female
Left auricle	mRNA-seq	>25 million reads per sample	3 male, 1 female
Right auricle	mRNA-seq	>25 million reads per sample	3 male, 2 female
Right ventricle	mRNA-seq	>25 million reads per sample	3 male, 3 female
Skeletal muscle (bicep)	mRNA-seq	>25 million reads per sample	3 male, 3 female

Supplementary Table 1. Details of tissues sequenced to generate the RNA-seq dataset for the cardiovascular gene expression atlas. Skeletal muscle (bicep) was also included, as an example of another muscle tissue, for comparative analysis. All libraries were Illumina 125 bp paired end stranded libraries.

Cluster	Highest expression	Total genes	Total ENSOARG ID[a]	Total pseudogenes	Total uncharacterised proteins	Total with no GO terms
**1**	Valves	3543	529	19	440	331
**3**	Valves	192	40	1	40	23
**4**	Bicep and myocardium	164	5	0	24	9
**13**	Bicep and left ventricle	48	0	1	13	10
**22**	Valves	27	5	0	5	1
**23**	Bicep	25	1	0	2	1
**24**	Valves	25	3	0	3	2
**28**	Bicep	24	2	0	1	1
**36**	Auricles	20	5	0	5	4
**66**	Left ventricle	15	0	0	2	2
**83**	Left ventricle	13	1	0	2	5
**90**	Left ventricle	13	1	0	2	2

Supplementary Table 2. Minimally annotated genes in clusters with defined expression patterns

[a.] These genes had not been allocated a gene symbol and were listed under their Ensembl gene name, starting with ENSOARG.

Gene symbol	Gene description	Disease	OMIM number
ANKH	Inorganic pyrophosphate transport regulator also known as progressive ankylosis protein homologue	Chondrocalcinosis 2	118600
BGN	Biglycan	Meester-Loeys (aortic aneurysm) syndrome; Spondyloepimetaphyseal dysplasia, X-linked	300989<br><br>300106
COL1A1	Collagen type 1, alpha 1	Osteogenesis imperfecta	166200, 166210, 166220, 259420
ENPP1	Ectonucleotide pyrophosphatase/ phosphodiesterase 1	Generalised arterial calcification of infancy	208000
FBN1	Fibrillin 1	Marfan syndrome	154700
FBN2	Fibrillin 2	Congenital contractural arachnodactyly	121050
MGP	Matrix gamma-carboxyglutamic acid, also known as matrix Gla protein	Keutel (ectopic calcification) syndrome	154870
MMP2	Matrix metalloproteinase 2	Multicentric osteolysis, nodulosis, and arthropathy	120360
NT5E	Ecto-5-prime nucleotidase	Calcification of joints or arteries	211800
RUNX2	Runt-related transcrirption factor 2	Cleidocranial dysplasia	119600
TNFRSF11B	Tumour necrosis factor receptor superfamily, member 11B, also known as osteoprotegerin	Paget disease of bone 5, juvenile-onset	239000
SPP1	Secreted phosphoprotein 1, also known as osteopontin	Expressed in calcified arteries; knockout mouse shows disordered wound healing and defective macrophage response to infection	
TIMP1	Tissue inhibitor of metalloproteinase 1	Knockout mice have altered vascular permeability and immune response	

Supplementary Table 3. diseases associated with genes examined in sheep cardiovascular tissues during development. OMIM - Online Mendelian Inheritance in Man (https://omim.org/)

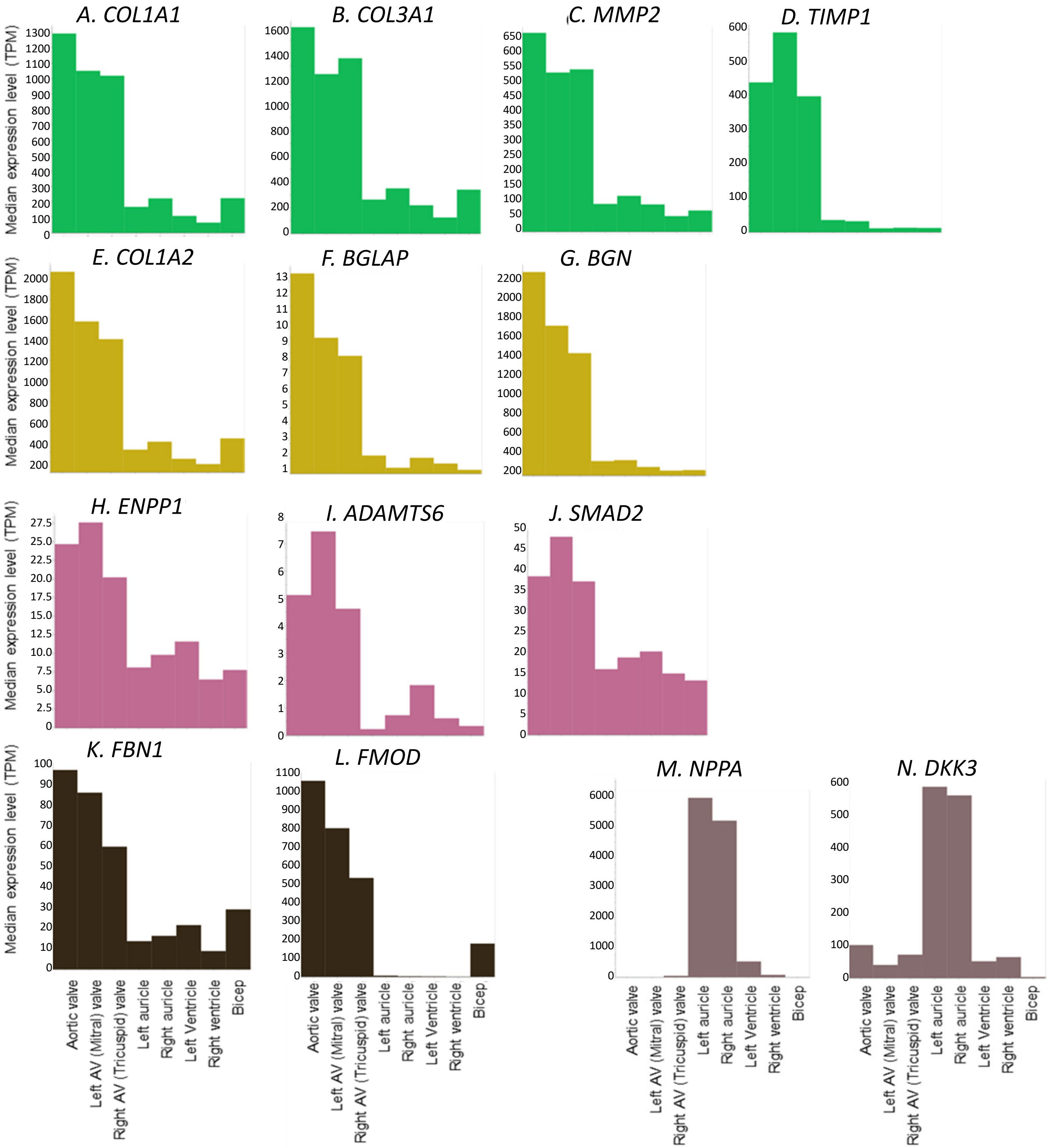

Supplementary Figure 1. RNA-seq expression profiles of selected genes. Expression levels were measured using RNA-seq and shown as median expression levels in transcripts per million (TPM; n = 4-6). Y axis shows normalised median TPM (Bush et al., 2017). (A-D) Cluster 1 gene expression profiles. Genes include COL1A1 (collagen type I alpha 1), COL3A1 (collagen type III alpha 1), MMP2 (matrix metalloproteinase 2) and TIMP1 (tissue inhibitor of metalloproteinases 1). (E-G) Cluster 3 gene expression profiles. Genes include COL1A2 (collagen type I alpha 2), BGLAP (bone gamma-carboxyglutamate protein) and BGN (biglycan). (H-J) Cluster 22 gene expression profiles. Genes include ENPP1 (ectonucleotide pyrophosphate/phosphodiesterase 1), ADAMTS6 (ADAM metallopeptidase with thrombospondin type 1 motif 6) and SMAD2 (SMAD family member 2). (K-L) Cluster 24 gene expression profiles. Genes include FBN1 (fibrillin 1) and FMOD (fibromodullin). (M-O) Cluster 36 gene expression profiles. Genes include NPPA (natriuretic peptide A) and DKK3 (Dickkopf WNT signalling pathway inhibitor 3).

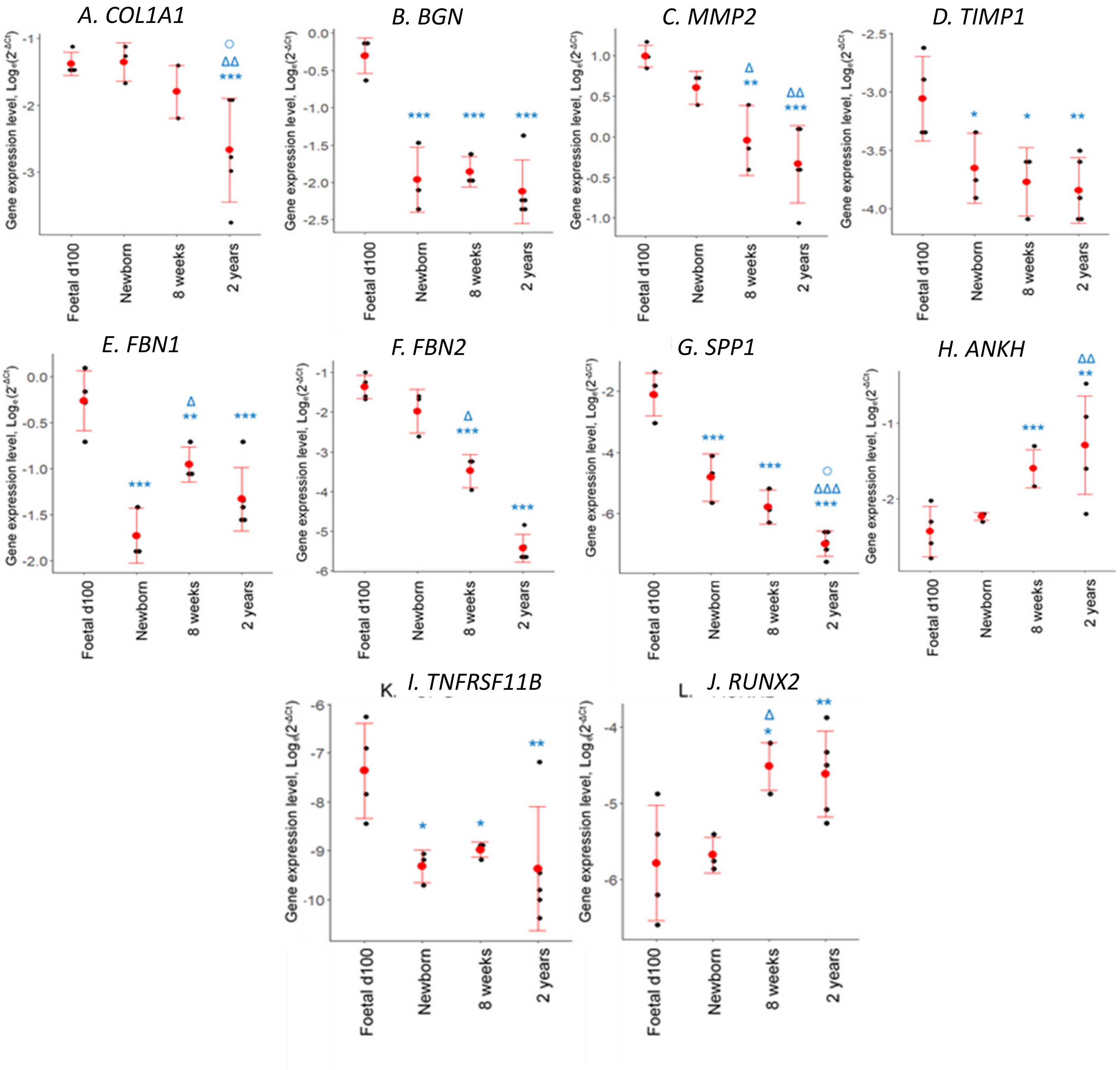

Supplementary Figure 2. Gene expression profiles during development in the left ventricle. Only those genes that showed a significant change are shown. Genes include: (A) collagen type I alpha 1, *COL1A1*, (B) biglycan, *BGN*, (C) matrix metalloproteinase 2, *MMP2*, (D) TIMP metallopeptidase inhibitor 1, *TIMP1*, (E) fibrillin 1, *FBN1*, (F) fibrillin 2, *FBN2*, (G) secreted phosphoprotein 1/osteopontin, *SPP1*, (H) progressive ankylosis protein, *ANKH*, (I) osteoprotegerin, *TNFRSF11B*, and (J) Runt-related transcription factor 2, *RUNX2*. Black dots show gene expression from individual animals (n = 3-5) and red dot and error bars show the mean ± standard deviation (SD) per tissue. Gene expression levels were normalised to the geomean of *GAPDH* and *YWHAZ*. In blue, asterisk (*) denotes significant differences compared to foetal d100 sheep, triangle (Δ) compared to newborn sheep and circle (o) compared to 8 week old sheep, where 1 symbol = $0.01<p<0.05$, 2 symbols = $0.001<p<0.01$ and 3 symbols = $p<0.001$.

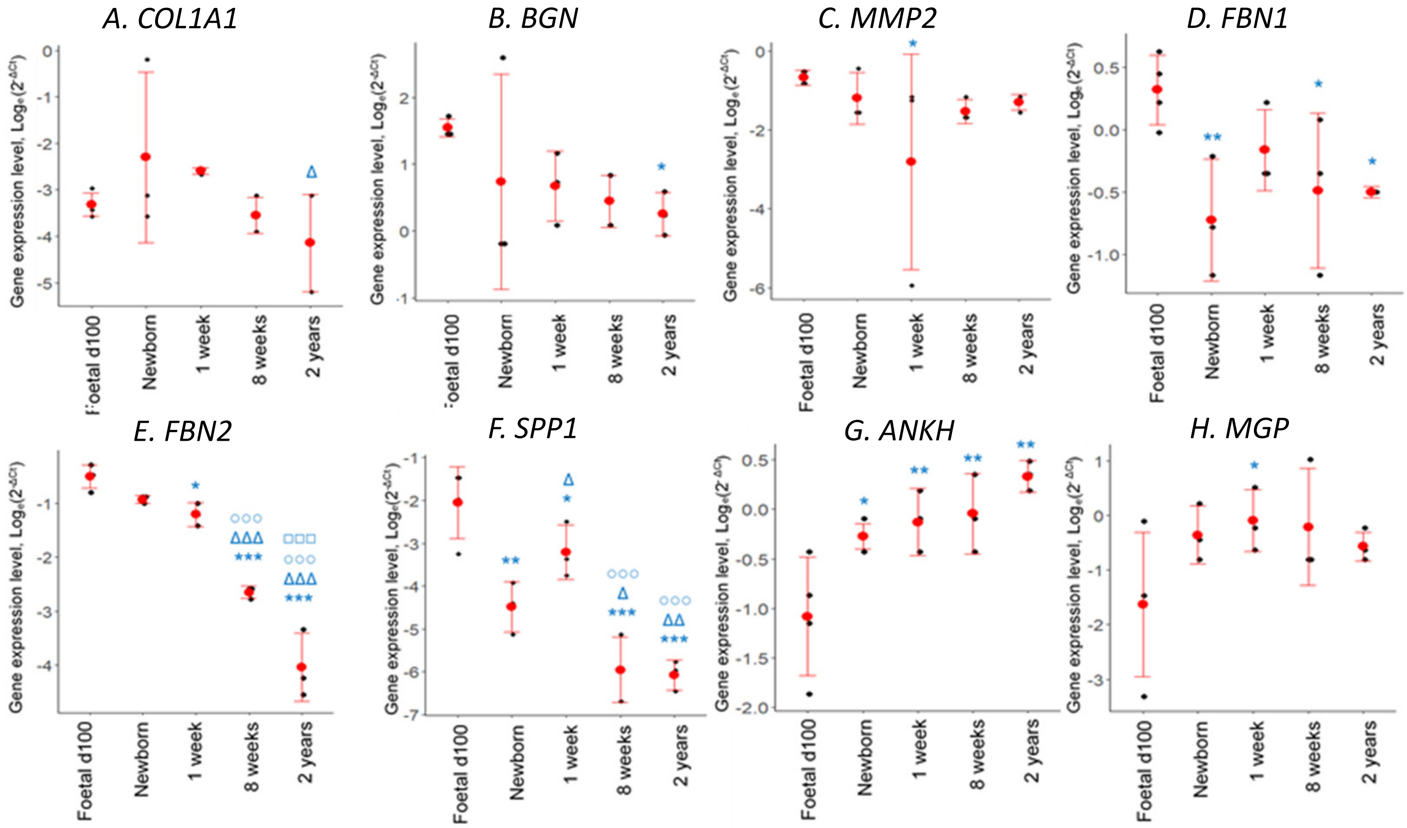

Supplementary Figure 3. Gene expression profiles during development in the interventricular septum. Only those genes that showed a significant change are shown. Genes include: (A) collagen type I alpha 1, *COL1A1*, (B) biglycan, *BGN*, (C) matrix metalloproteinase 2, *MMP2*, (D) fibrillin 1, *FBN1*, (E) fibrillin 2, *FBN2*, (F) secreted phosphoprotein 1/osteopontin, *SPP1*, (G) progressive ankylosis protein, *ANKH*, and (H) matrix Gla protein, *MGP*. Black dots show gene expression from individual animals (n = 3-5) and red dot and error bars show the mean ± standard deviation (SD) per tissue. Gene expression levels were normalised to the geomean of *GAPDH* and *YWHAZ*. In blue, asterisk (*) denotes significant differences compared to foetal d100 sheep, triangle (Δ) compared to newborn sheep, circle (o) compared to 1 week old sheep and square (□) compared to 8 week old sheep, where 1 symbol = $0.01<p<0.05$, 2 symbols = $0.001<p<0.01$ and 3 symbols = $p<0.001$.

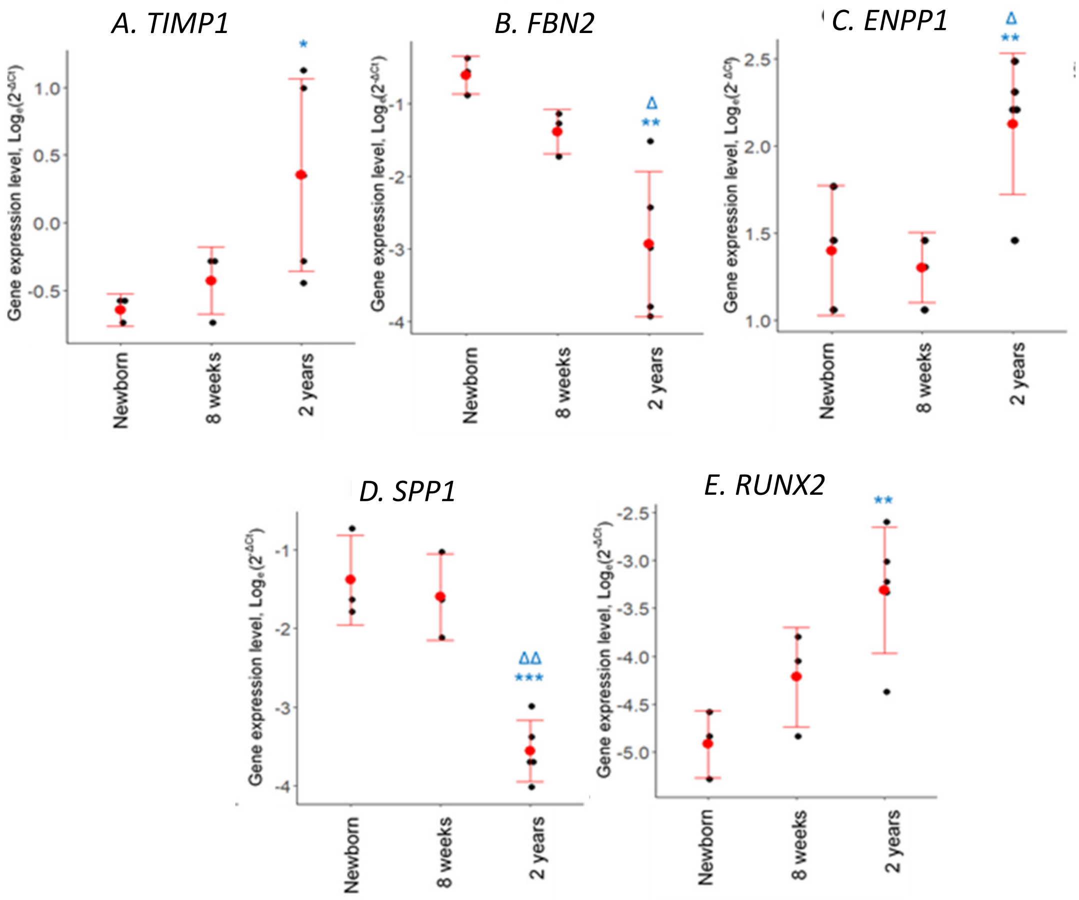

Supplementary Figure 4. Gene expression profiles during development in the pulmonary artery. Only those genes that showed a significant change are shown. Genes include: (A) TIMP metallopeptidase inhibitor 1, *TIMP1*, (B) fibrillin 2, *FBN2*, (C) ectonucleotide pyrophosphatase/ phosphodiesterase 1, *ENPP1*, (D) secreted phosphoprotein 1/osteopontin, *SPP1*, and (E) Runt-related transcription factor 2, *RUNX2*. Black dots show gene expression from individual animals (n = 3-5) and red dot and error bars show the mean ± standard deviation (SD) per tissue. Gene expression levels were normalised to the geomean of *GAPDH* and *YWHAZ*. In blue, asterisk (*) denotes significant differences compared to newborn sheep, triangle (Δ) compared to 8 week old sheep, where 1 symbol = $0.01<p<0.05$, 2 symbols = $0.001<p<0.01$ and 3 symbols = $p<0.001$.

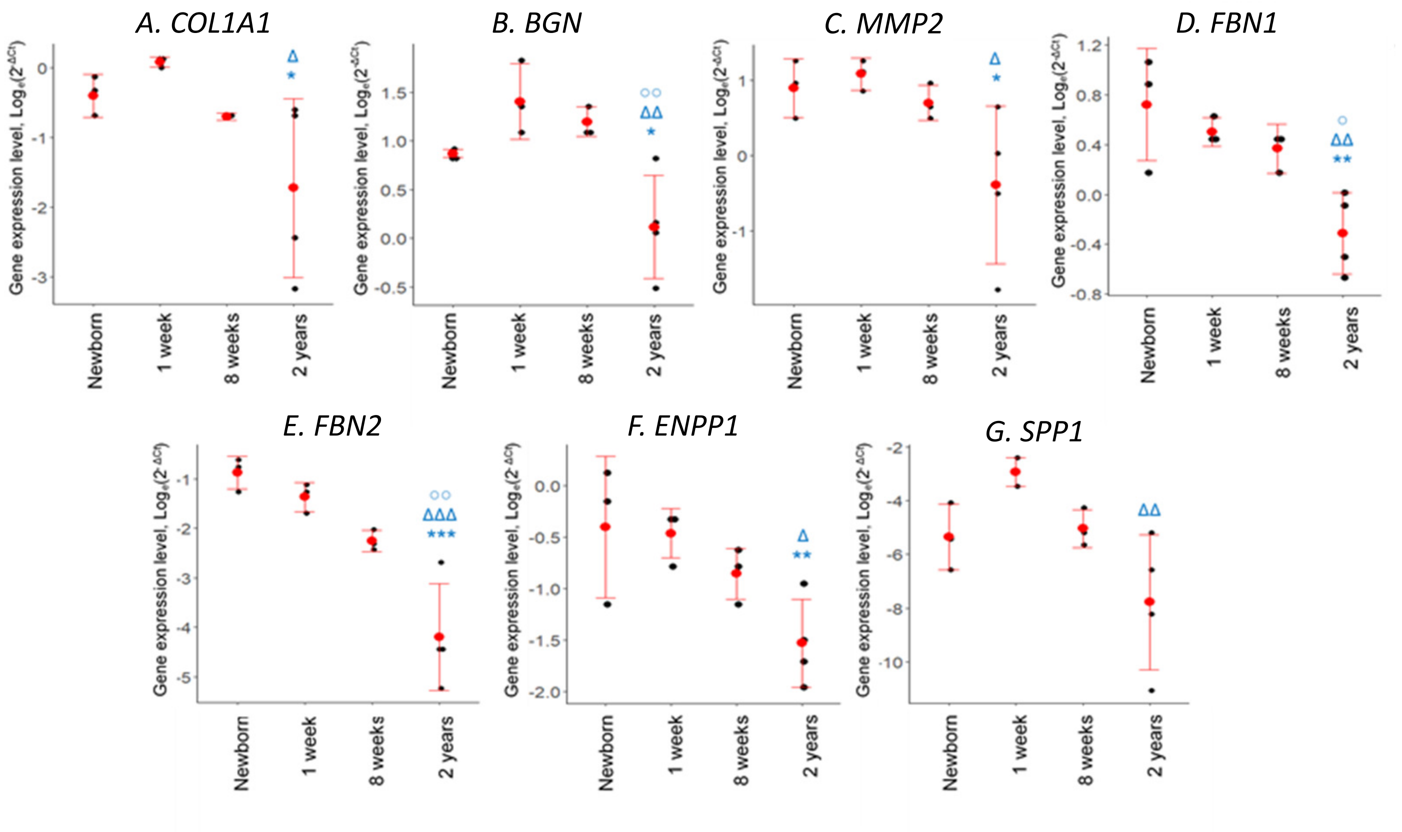

Supplementary Figure 5. Gene expression profiles during development in the aortic root. Only those genes that showed a significant change are shown. Genes include: (A) collagen type I alpha 1, *COL1A1*, (B) biglycan, *BGN*, (C) matrix metalloproteinase 2, *MMP2*, (D) fibrillin 1, *FBN1*, (E) fibrillin 2, *FBN2*, (F) ectonucleotide pyrophosphatase/ phosphodiesterase 1, *ENPP1*, and (G) secreted phosphoprotein1/osteopontin. Black dots show gene expression from individual animals (n = 3-5) and red dot and error bars show the mean ± standard deviation (SD) per tissue. Gene expression levels were normalised to the geomean of *GAPDH* and *YWHAZ*. In blue, asterisk (*) denotes significant differences compared to newborn sheep, triangle (Δ) compared to 1 week old sheep and circle (o) compared to 8 week old sheep, where 1 symbol = $0.01<p<0.05$, 2 symbols = $0.001<p<0.01$ and 3 symbols = $p<0.001$.

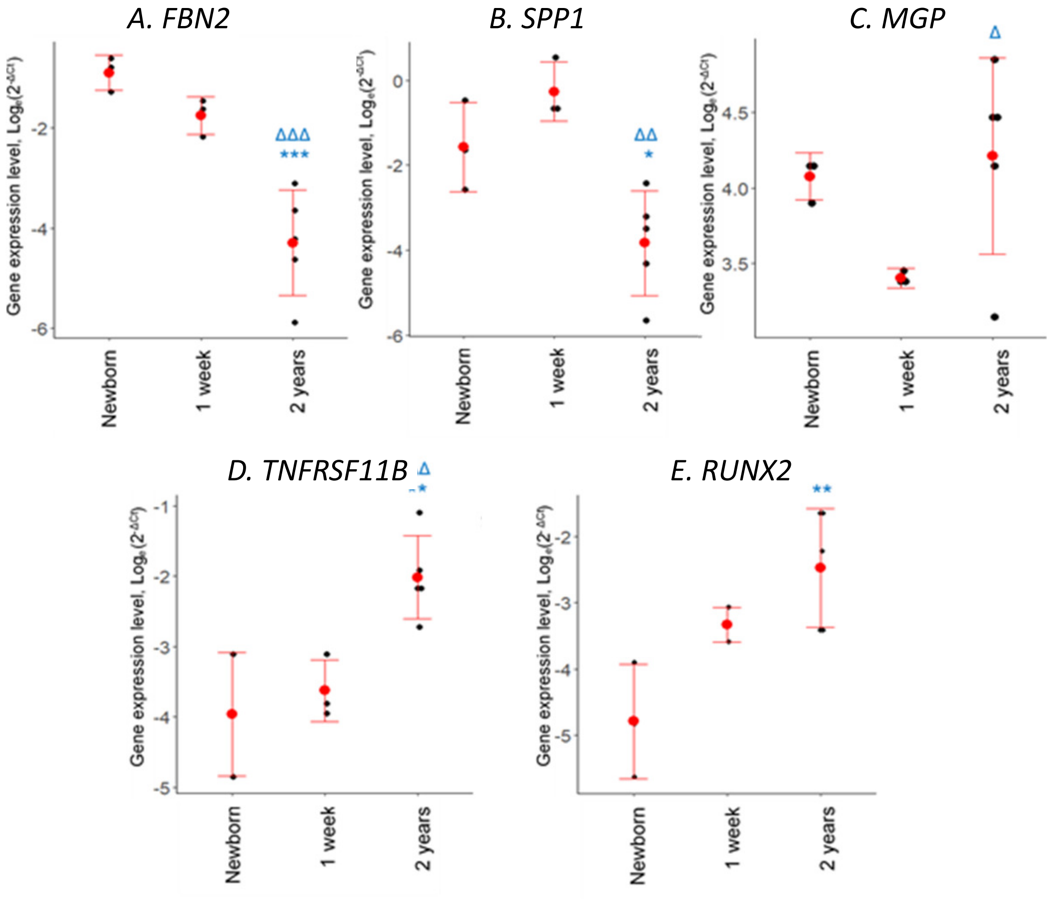

Supplementary Figure 6. Gene expression profiles during development in the aortic arch. Only those genes that showed a significant change are shown. Genes include: (A) fibrillin 2, *FBN2*, (B) secreted phosphoprotein1/osteopontin, *SPP1*, (C) matrix Gla protein, *MGP*, (D) osteoprotegerin, *TNFRSF11B*, and (E) Runt-related transcription factor 2, *RUNX2*. Black dots show gene expression from individual animals (n = 3-5) and red dot and error bars show the mean ± standard deviation (SD) per tissue. Gene expression levels were normalised to the geomean of *GAPDH* and *YWHAZ*. In blue, asterisk (*) denotes significant differences compared to newborn sheep and triangle (Δ) compared to 1 week old sheep, where 1 symbol = 0.01<p<0.05, 2 symbols = 0.001<p<0.01 and 3 symbols = p<0.001.

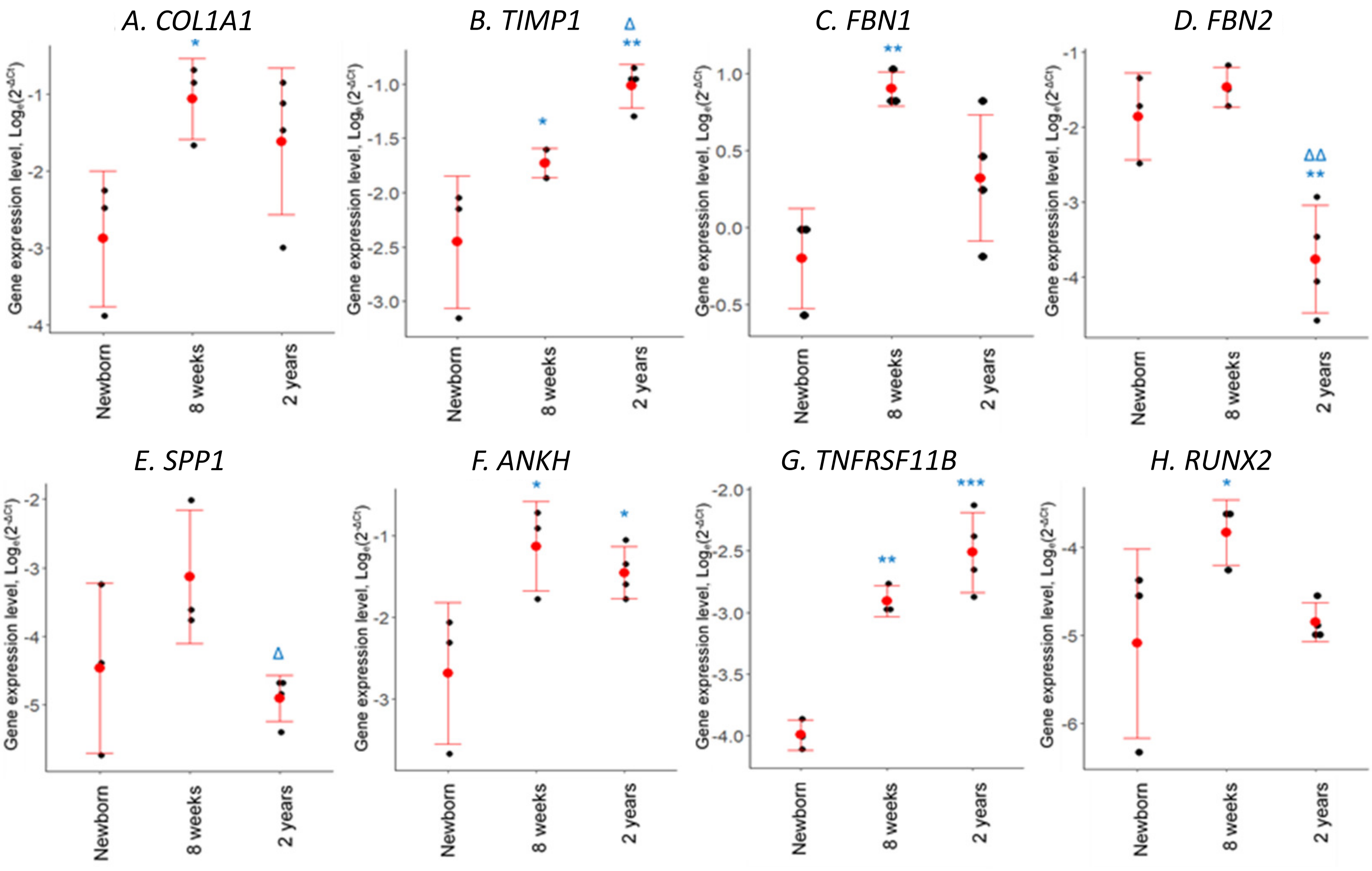

Supplementary Figure 7. Gene expression profiles during development in the abdominal aorta. Only those genes that showed a significant change are shown. Genes include: (A) collagen type I alpha 1, *COL1A1*, (B) TIMP metallopeptidase inhibitor 1, *TIMP1*, (C) fibrillin 1, *FBN1*, (D) fibrillin 2, *FBN2*, (E) secreted phosphoprotein 1/osteopontin, *SPP1*, (F) progressive ankylosis protein, *ANKH*, (G) osteoprotegerin, *TNFRSF11B*, and (H) Runt-related transcription factor 2, *RUNX2*. Black dots show gene expression from individual animals (n = 3-5) and red dot and error bars show the mean ± standard deviation (SD) per tissue. Gene expression levels were normalised to the geomean of *GAPDH* and *YWHAZ*. In blue, asterisk (*) denotes significant differences compared to newborn sheep and triangle (Δ) compared to 8 week old sheep, where 1 symbol = $0.01<p<0.05$, 2 symbols = $0.001<p<0.01$ and 3 symbols = $p<0.001$.

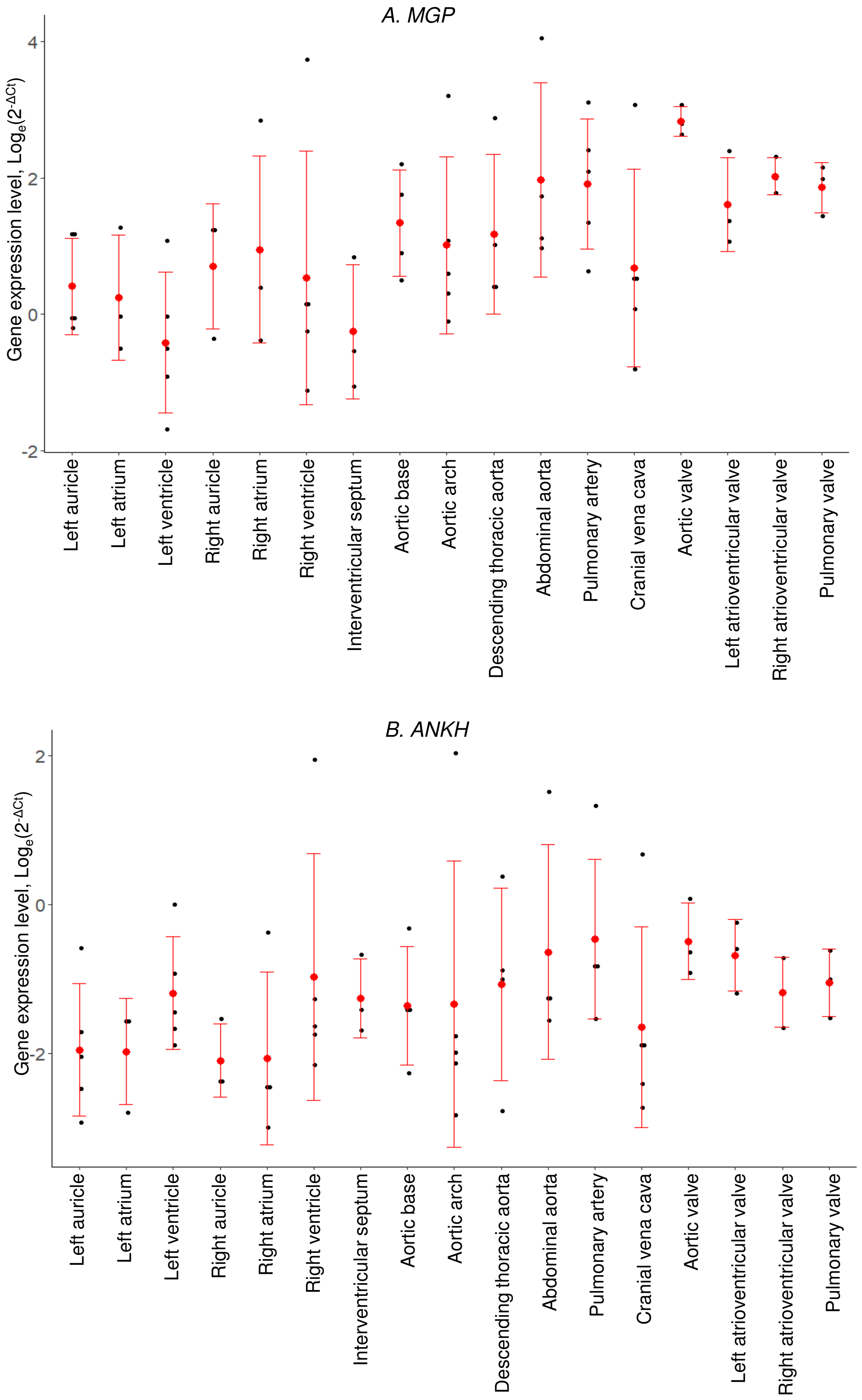

Supplementary Figure 8. mRNA expression profile for (A) matrix Gla protein (*MGP*), (B) progressive ankylosis protein homologue (ANKH). Gene expression levels were normalised to the geomean of *GAPDH* and *YWHAZ*. Dot plots show individual data points (black dot), the mean expression for each tissue (red dot) and standard deviation (red error bars).

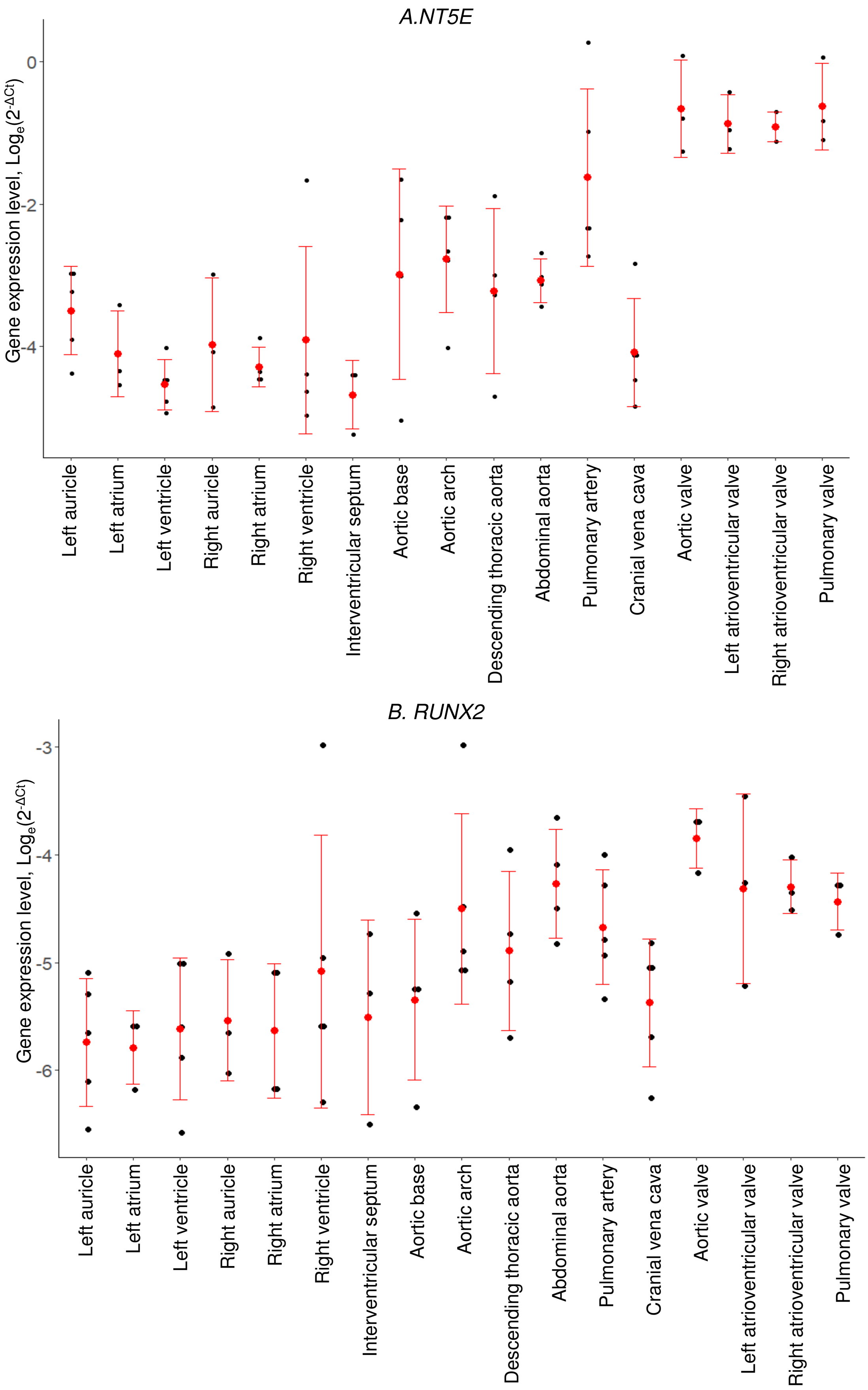

Supplementary Figure 9. mRNA expression profile for (A) ecto-5'-nucleotidase (*NT5E*) and (B) Runt-related transcription factor 2 (*RUNX2*). Gene expression levels were normalised to the geomean of *GAPDH* and *YWHAZ*. Dot plots show individual data points (black dot), the mean expression for each tissue (red dot) and standard deviation (red error bars).

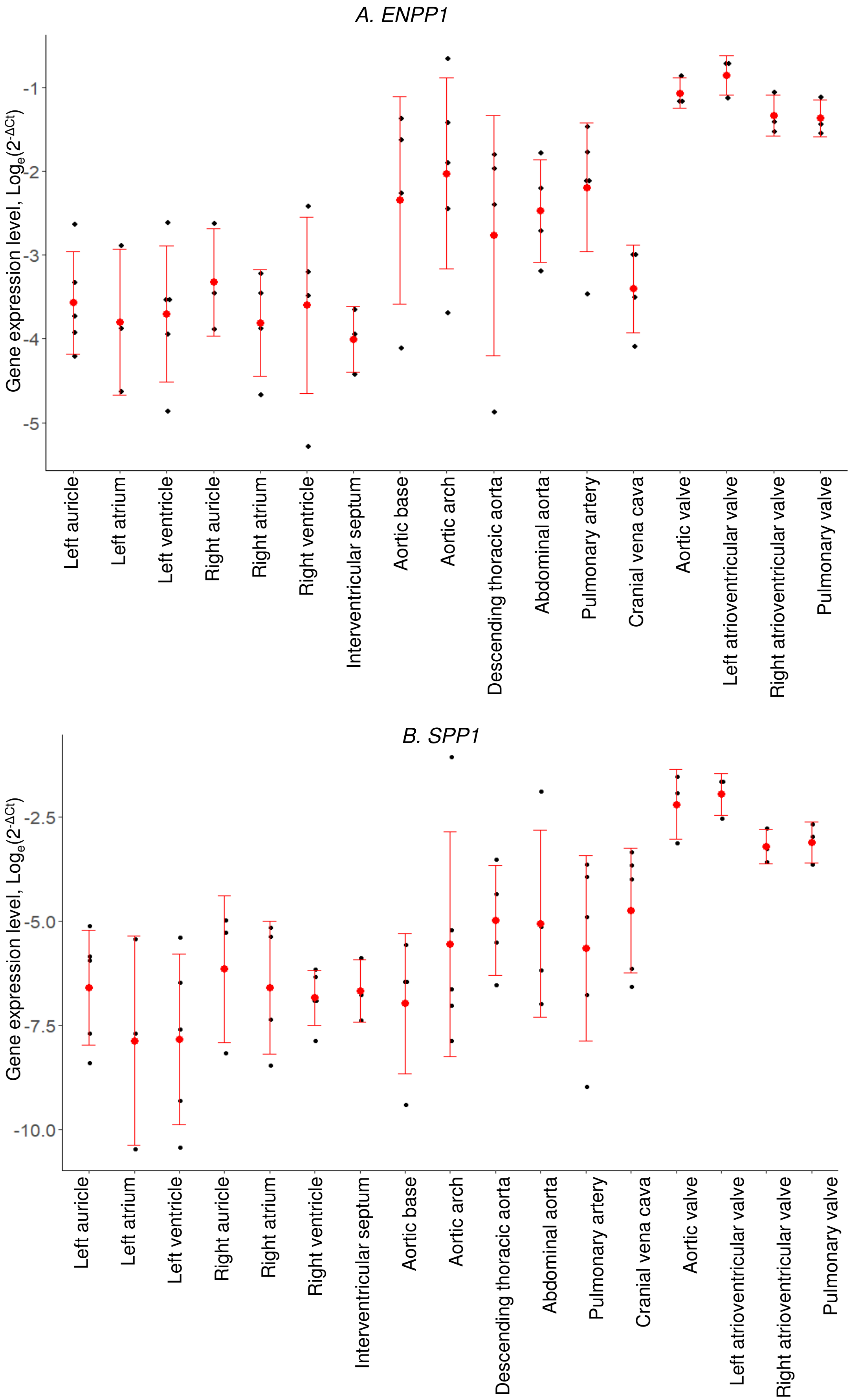

Supplementary Figure 10. mRNA expression profile for (A) ectonucleotide pyrophosphatase/phosphodiesterase 1 (*ENPP1*) and (B) secreted phosphoprotein 1/osteopontin (*SPP1*). Gene expression levels were normalised to the geomean of *GAPDH* and *YWHAZ*. Dot plots show individual data points (black dot), the mean expression for each tissue (red dot) and standard deviation (red error bars).
